# Citrullination of AKT2 Catalyzed by PAD1 Facilitates the Maintenance of Stemness Characteristics of Ovarian Cancer Stem‐Like Cells in Ovarian Cancer

**DOI:** 10.1002/advs.202501014

**Published:** 2025-08-22

**Authors:** Teng Xue, Xiaoqiu Liu, Chao Song, Shujia Fei, Jian Gu, Yun Han, Jia Xing, Xiaohan Liu, Fei Liang, Paul R Thompson, Xuesen Zhang

**Affiliations:** ^1^ Department of Histology and Embryology College of Basic Medical Science China Medical University Shenyang 110122 China; ^2^ Department of Pathology College of Basic Medical Science China Medical University Shenyang 110122 China; ^3^ State Key Laboratory of Reproductive Medicine Nanjing Medical University Nanjing 211166 China; ^4^ Department of Obstetrics and Gynecology Affiliated Hospital 2 of Nantong University and Nantong First People's Hospital Nantong Jiangsu 226001 China; ^5^ Department of Biochemistry and Molecular Pharmacology University of Massachusetts Medical School Worcester MA 01655 USA

**Keywords:** AKT2, citrullination, ovarian cancer stem‐like cells, PAD1, stemness maintenance

## Abstract

The presence of ovarian cancer stem‐like cells (OCSLCs) is a crucial driving force for malignant progression, metastasis, and tumor recurrence of ovarian cancer (OC). However, the mechanisms underlying activation and maintenance of OCSLC stemness remain unclear. Here, it is identified an increased expression of peptidylarginine deiminase 1 (PAD1) in OC cells, particularly within the CD133^+^ subset or cisplatin‐resistant cells, which is positively associated with the upregulation of stemness‐related markers and maintenance of stemness in OCSLCs. Mechanistically, PAD1 specifically binds to the kinase domain of AKT2 and catalyzes citrullination at R202. Citrullination subsequently promotes AKT2 phosphorylation at S474 and T309, the two critical residues for AKT2 kinase activity. As a major driver of OC malignancy, the activation of AKT2 leads to an increased expression of CCAAT/Enhancer Binding Protein Beta (CEBPβ), thereby promoting CEBPβ enrichment to the promoters of a subset of stemness‐related genes. Moreover, PAD1 gene silencing, inhibition of AKT2 citrullination, and AKT2 mutation all decrease the tumor‐initiating ability of OC cells both in vitro and in vivo. Importantly, treatment with a PAD1 inhibitor can resensitize cisplatin‐resistant OC cells to cisplatin treatment, suggesting that targeting PAD1/AKT2/CEBPβ signaling axis in OCSLCs may be highly effective in preventing OC progression.

## Graphical abstract

PAD1 is upregulated within the CD133^+^ subset or cisplatin‐resistant ovarian cancer cells, which is positively associated with the upregulation of stemness‐related markers and maintenance of stemness in OCSLCs. Mechanistically, PAD1‐catalyzed AKT2 citrullination at R202 facilitates AKT2 phosphorylation, promoting CEBPβ expression and its binding to the promoters of the cancer stemness‐related genes, leading to gene transactivation.

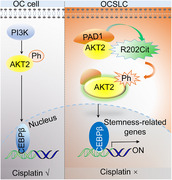

## 1 Introduction

Epithelial ovarian cancer (referred to as ovarian cancer, OC) is one of the three major malignant tumors in the female reproductive system, with a poor prognosis and the highest mortality rate among gynecological tumors.^[^ ^1^ ^]^ Currently, eradication surgery and chemotherapy based on platinum combined with paclitaxel are considered the primary treatment modalities for OC.^[^ ^2^ ^]^ However, a vast majority of patients experience tumor recurrence and develop drug resistance, which ultimately leads to treatment failure.^[^ ^3^, ^4^ ^]^ Cancer stem cells (CSCs) are a small subset of drug‐resistant tumor cells that drive malignant progression, metastasis, and tumor recurrence.^[^ ^5^, ^6^ ^]^ Similar to other strategies against tumor resistance, OC treatments should focus in effectively targeting and eliminating ovarian cancer stem‐like cells (OCSLCs).^[^ ^7^, ^8^ ^]^ Therefore, a comprehensive understanding of the mechanisms involved in the activation and maintenance of OCSLCs is imperative for the success of this therapeutic approach.

AKT is located at the intersection of various cancer signaling pathways and ultimately plays a crucial role in governing tumor cell fates, including CSC behavior and function.^[^ ^9^, ^10^ ^]^ Inhibiting the PI3K/AKT pathway may lead to decreased proliferation of cancer stem‐like cells, increased apoptosis, and reduced expression of stem cell‐related markers, such as CD133, OCT4, SOX2, and NANOG.^[^ ^10^, ^11^ ^]^ Additionally, most of the upstream regulators and downstream mediators of the AKT pathway are encoded by oncogenes or tumor suppressor genes whose loss or activation, respectively, accelerate tumorigenesis.^[^ ^12^, ^13^ ^]^ Therefore, the nodal points within the PI3K/AKT signaling pathway provide potential targets for application in targeted cancer therapy. As one of the most extensively studied signaling pathways in OC, alterations in the PI3K/AKT pathway are reported to be essential for maintaining OCSLC stemness and acquiring drug resistance.^[^ ^14^, ^15^ ^]^ The utilization of inhibitors targeting PI3K/AKT can partially reverse chemotherapy resistance including that to cisplatin in OC cells.^[^ ^16^ ^]^ Although PI3K/AKT inhibitors offer new options for clinical cancer treatment, these drugs also exhibit substantial side effects when used against cancer cells. Consequently, it is extremely challenging to develop an ideal AKT inhibitor with low toxicity while maintaining high efficacy. In addition, the high homology among the three AKT subtypes limits the study and development of subtype‐specific inhibitors, which may alleviate their toxic load.^[^ ^17^ ^]^ Hence, strategies involving the development of novel AKT inhibitors or a combination of AKT inhibitors with other anti‐cancer drugs could solve this therapeutic dilemma.

Citrullination is a complex and emerging post‐translational modification (PTM) that has garnered increasing attention in recent years. It refers to the enzymatic conversion of positively charged arginine residues into citrulline, catalyzed by peptidylarginine deiminases (PADs) in the presence of Ca^2+^.^[^ ^18^ ^]^ By altering the charge distribution of substrate proteins, citrullination can modulate both protein structure and function. The initial evidence establishing a correlation between citrullination and stemness was derived from PAD4, which regulates the expression of c‐Myc in bone marrow cells and inhibits the proliferation of multipotent hematopoietic stem cells.^[^ ^19^ ^]^ PAD4 also catalyzes the citrullination of histone H1, leading to an open chromatin structure that is necessary for efficient stem cell reprogramming in early mouse embryos.^[^ ^20^ ^]^ Importantly, PAD4 has a novel tumor suppressor role in breast cancer stem cells, where it downregulates the expression of the stemness master transcription factors NANOG and OCT4 in the CSC compartment.^[^ ^21^ ^]^ The close link between PAD4‐catalyzed citrullination and stemness maintenance promoted us to hypothesize that such an association also exists in OC. To address this question, we examined the expression of PADs in OC tissues and OC cell lines, and found that PAD1 was specifically upregulated in OCSLCs. We then explored the function of PAD1 in ovarian carcinogenesis and chemoresistance, with a particular focus on elucidating the molecular mechanism by which PAD1‐mediated AKT2 citrullination regulates the maintenance of stemness in OCSLCs within OC.

## 2 Results

### 2.1 PAD1 Expression is Positively Associated with OC Progression

**Figure 1:**
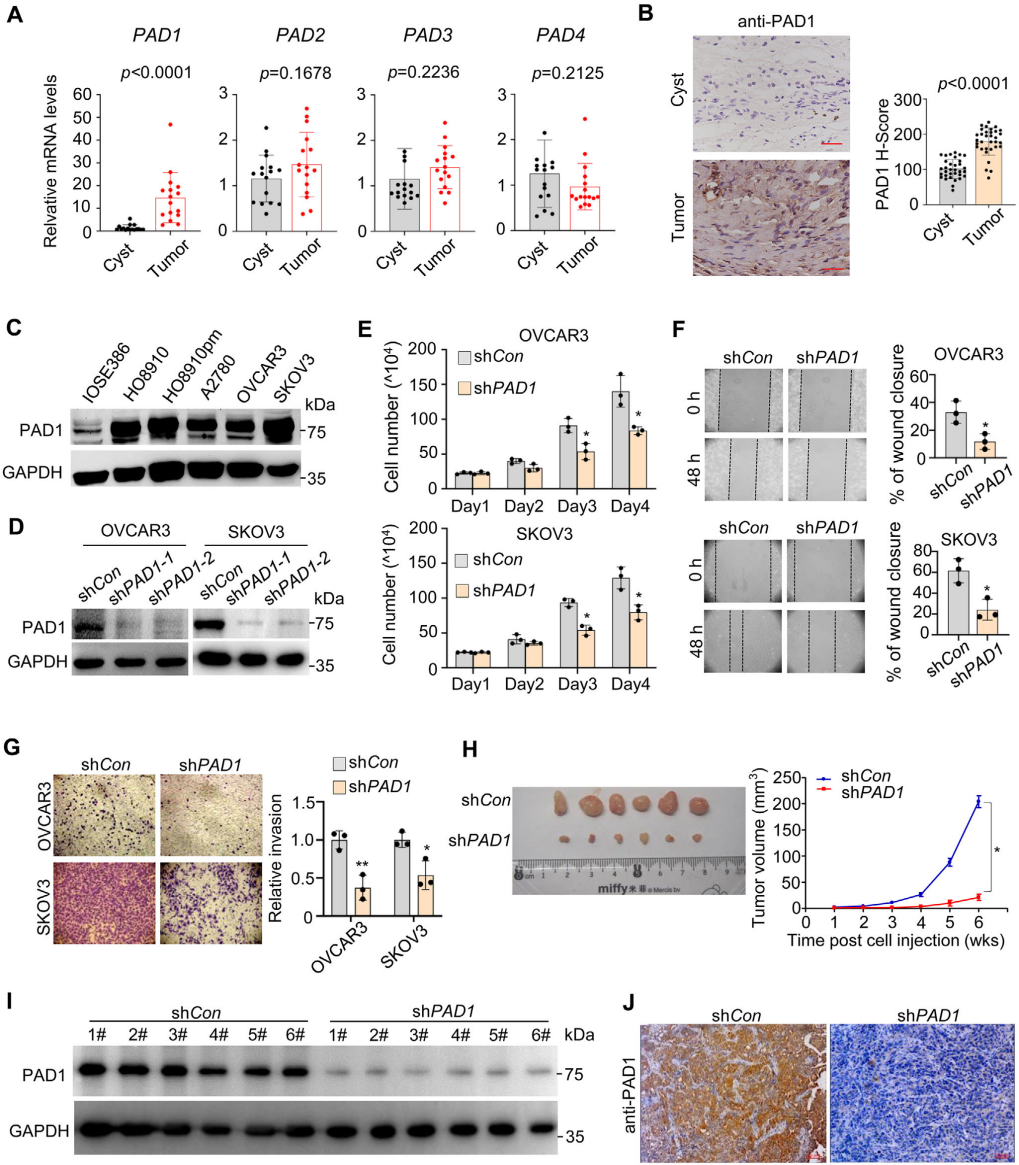
PAD1 expression is positively associated with OC progression A) RT‐qPCR analysis of PADs expression in tissues from clinical ovarian cancer (n = 16) and ovarian cyst (n = 16). *GAPDH* was used as the reference control. B) Left: Expression levels of PAD1 were examined by immunohistochemistry staining in representative sections from ovarian cancer patients (n = 33) and ovarian cyst section (n = 33). Bar = 20 µm. Right: Statistical analysis of H‐Score (histochemical score) was conducted to evaluate PAD1 expression levels in both ovarian cancer and cyst tissues. C) Western blot analysis of PAD1 expression in normal ovarian epithelial cell lines IOSE386 and different ovarian cancer cell lines. GAPDH served as the loading control. D) Analysis for PAD1 protein expression in PAD1 stable knockdown OVCAR3 and SKOV3 cells. GAPDH served as loading control. E) PAD1 KD OVCAR3 cells (top) and PAD1 KD SKOV3 cells (bottom) were cultured in regular medium, at indicated times, and cell numbers were counted under light microscope. Compared to the respective shRNA control cells. F) Representative images at 48 h after scratching (left) and quantification (right) for wound healing assay in PAD1 KD OVCAR3 cells (top), and PAD1 KD SKOV3 cells (bottom), compared to the respective shRNA control cells. G) Representative images (left) and quantification (right) for transwell assay in PAD1 KD OVCAR3 (top) and PAD1 KD SKOV3 cells (bottom), compared to the respective shRNA control cells. H) Dissected tumors collected from nude mice (n = 6) injected with shRNA control OVCAR3 cells on the left flank (sh*Con*), and stable *PAD1* depletion OVCAR3 cells on the right flank (sh*PAD1*) at the experimental endpoint (Left). The volume of tumors at indicated time after implantation of *PAD1* depletion cells or sh*Con* cells in nude mice (Right). I) Protein levels of PAD1 in the lysates from tumor tissues (H). GAPDH served as a loading control. J) Immunohistochemistry analysis of PAD1 in representative sections from tumor tissues (H). Results are presented as mean ± SD, n = 3. *p < 0.05, **p < 0.01. A, B, E, F, G, H: Student's *t*‐test.

To investigate the expression profiles of distinct PADs in OC, we collected clinical OC tissues along with non‐cancerous tissues from ovarian cysts and analyzed them by quantitative real‐time PCR (RT‐qPCR). The results showed a significant increase in *PAD1* expression in OC tissues compared to that in ovarian cysts, whereas there was no such difference for other *PADs* (**Figure** **1**A). To validate these findings, we examined the PAD1 protein expression levels in clinical OC tissues using immunohistochemistry. Tumor sections showed strong positive signals for PAD1, while less intense signals were observed in cyst sections (representative image shown in Figure 1B). These findings suggested an involvement of PAD1 in OC tumorigenesis. To test this hypothesis, we compared PAD1 expression in different OC cell lines with that in non‐tumorigenic ovarian epithelial IOSE386 cells. Consistent with our clinical findings, PAD1 protein levels were higher in a range of OC cell lines than in IOSE386 cells (Figure 1C). We next generated PAD1‐depleted human ovarian cancer cell lines from OVCAR3 and SKOV3 cells using a lentiviral approach, and the efficiency of PAD1 knockdown was confirmed by western blot and RT‐qPCR (Figure 1D, Figure S1A, Supporting Information). The depletion of PAD1 had no significant effect on the expression levels of other PAD family members (Figure S1B–E, Supporting Information). Notably, PAD1 depletion significantly suppressed OC cell proliferation (Figure 1E), migration (Figure 1F), and invasion (Figure 1G). Moreover, we validated the in vitro phenotype of PAD1‐depleted OVCAR3 cells using a xenograft mouse model. Nude mice inoculated subcutaneously with PAD1 knockdown cells exhibited smaller tumors than those inoculated with shRNA control cells (Figure 1H). We further confirmed the efficiency of PAD1 knockdown in established tumors using western blot analysis and immunohistochemistry. As expected, tumors in mice harboring PAD1 KD cell lines exhibited diminished levels of PAD1 expression (Figure 1I,J). Additionally, the intraperitoneal metastatic xenograft mouse model demonstrated that PAD1 depletion significantly reduced both the number of metastatic implants (Figure S1F, Supporting Information) and the volume of ascites compared with control cells (Figure S1G, Supporting Information). These findings indicate that PAD1 is essential for OC cell proliferation, migration, and invasion.

### 2.2 PAD1 Increases Tumor Initiating Ability of OC Cells

**Figure 2:**
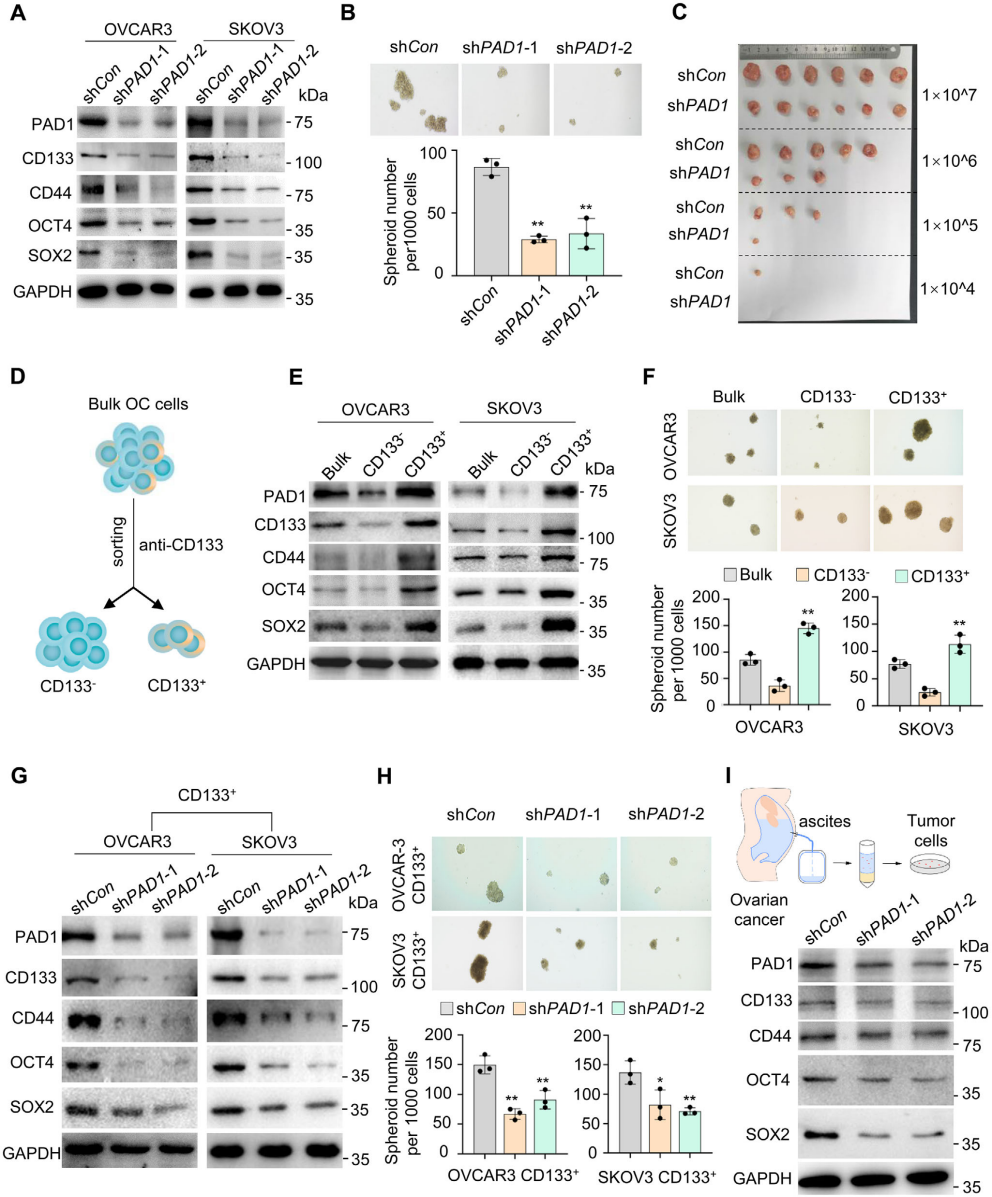
PAD1 increases tumor initiating ability of OC cells A) Western blot analysis of PAD1, CD133, CD44, OCT4, and SOX2 in PAD1 KD OVCAR3 (left) and PAD1 KD SKOV3 cells (right). GAPDH served as the loading control. B) Representative images of OVCAR3‐derived spheroids with diameters greater than 100 µm cultured in stem cell culture medium in flat bottom ultra‐low attachment plates without FBS (top) and quantification (bottom) upon PAD1 knockdown. C) Dissected tumors collected from nude mice injected with shRNA control OVCAR3 cells on the left flank (sh*Con*), and stable PAD1 depletion cells on the right flank (sh*PAD1*). D) Schematic diagram of sorting CD133^+^ OVCAR3 cells by flow cytometry. E) Western blot analysis of PAD1, CD133, CD44, OCT4, and SOX2 expression in CD133^−^ or CD133^+^ ovarian cancer cells. GAPDH served as the loading control. F) Representative images of OVCAR3‐ or SKOV3‐derived spheroids with diameters greater than 100 µm cultured in stem cell culture medium in flat bottom ultra‐low attachment plates without FBS (top) and quantification (bottom) in CD133^−^ and CD133^+^ cells. G) Western blot analysis of PAD1, CD133, CD44, OCT4, and SOX2 expression in CD133^+^ OVCAR3 and SKOV3 cells upon PAD1 knockdown. GAPDH served as the loading control. H) Representative images of OVCAR3‐ or SKOV3‐derived spheroids with diameters greater than 100 µm cultured in stem cell culture medium in flat bottom ultra‐low attachment plates without FBS (top) and quantification (bottom) upon PAD1 knockdown in CD133^+^ cells. I) Top: Schematic diagram of isolation and culture of tumor cells from ascitic fluid of ovarian cancer patients. Bottom: Western blot analysis of PAD1, CD133, CD44, OCT4 and SOX2 in PAD1‐knockdown tumor cells from ascite of ovarian cancer patients. GAPDH was used as the reference control. Results are presented as mean ± SD, n = 3. *p <0.05, **p < 0.01. B, F, H: one‐way ANOVA.

It has been demonstrated that stemness‐related markers, such as SOX2, OCT4, and CD133, can directly induce the stemness of embryonic or adult stem cells. Under physiological conditions, the genes encoding these proteins are finely regulated, whereas in the presence of tumors, abnormal activation of such genes occurs due to signaling pathway disorders, leading to the uncontrolled malignant growth and development of CSCs.^[^ ^9^, ^22^ ^]^ Given that OCSLCs determine the malignant progression of OC, and that tumor stemness maintenance is often driven by the expression of stemness‐related factors, we first examined the relationship between PAD1 and the expression of CSC markers. PAD1 knockdown resulted in decreased expression of CD133, CD44, OCT4, and SOX2 in both OVCAR3 and SKOV3 cells (**Figure** **2**A). Next, a tumor spheroid formation assay was employed to measure the amount of cancer stem‐like cells within the OVCAR3 cell line, which correlates with cancer metastasis and aggressiveness. The results demonstrated that depletion of PAD1 significantly inhibited tumorsphere formation efficiency (Figure 2B). Furthermore, we performed an extreme limiting dilution assay (ELDA) in nude mice with the parental control and PAD1 knockdown OVCAR3 cells to confirm the reduced OCSLC frequency upon PAD1 knockdown, since the gold‐standard functional in vivo assay for defining the self‐renewal of CSCs involves serial transplantation of isolated single cancer cells into immunocompromised mice. As expected, tumor incidence at all four dilutions tested (Figure 2C) and the relative CSLC frequency of PAD1 KD cells (Figure S2A, Supporting Information) were significantly lower than sh*Con* cells. Additionally, the tumors in mice inoculated with PAD1 KD cell lines exhibited decreased expression of CSC markers (Figure S2B, Supporting Information). These results suggest that endogenous PAD1 restricts the size of the OCSLC population within OC.

To test this hypothesis, we used CD133 as a cell surface marker to identify and isolate OCSLCs from bulk OC cells (Figure 2D, Figure S2C, Supporting Information). CD133^+^ cells isolated from both OC cell lines not only showed higher expression levels of PAD1 and stemness‐related markers (Figure 2E), but also exhibited a stronger ability for tumorsphere formation than CD133^−^ or bulk cells (Figure 2F). Upon PAD1 depletion in the two lines of CD133^+^ OCSLCs cells, the expression levels of stemness‐related markers significantly decreased (Figure 2G). Additionally, PAD1 depletion notably inhibited tumorsphere formation and invasion by these cells (Figure 2H, Figure S2D, Supporting Information). Given that OCSLCs are readily enriched in the ascites of patients with advanced OC, we isolated tumor cells from the ascites of two patients with OC. As expected, knockdown of PAD1 in these ascetic tumor cells also reduced the expression of CD133, CD44, OCT4 and SOX2 and significantly inhibited tumorsphere formation and cell invasion (Figure 2I, Figure S2E,F, Supporting Information). Taken together, these results suggest that PAD1 is involved in the maintenance of OCSLC stemness.

### 2.3 PAD1 Maintains the Stemness of OCSLCs through the Activation of AKT Signaling

**Figure 3:**
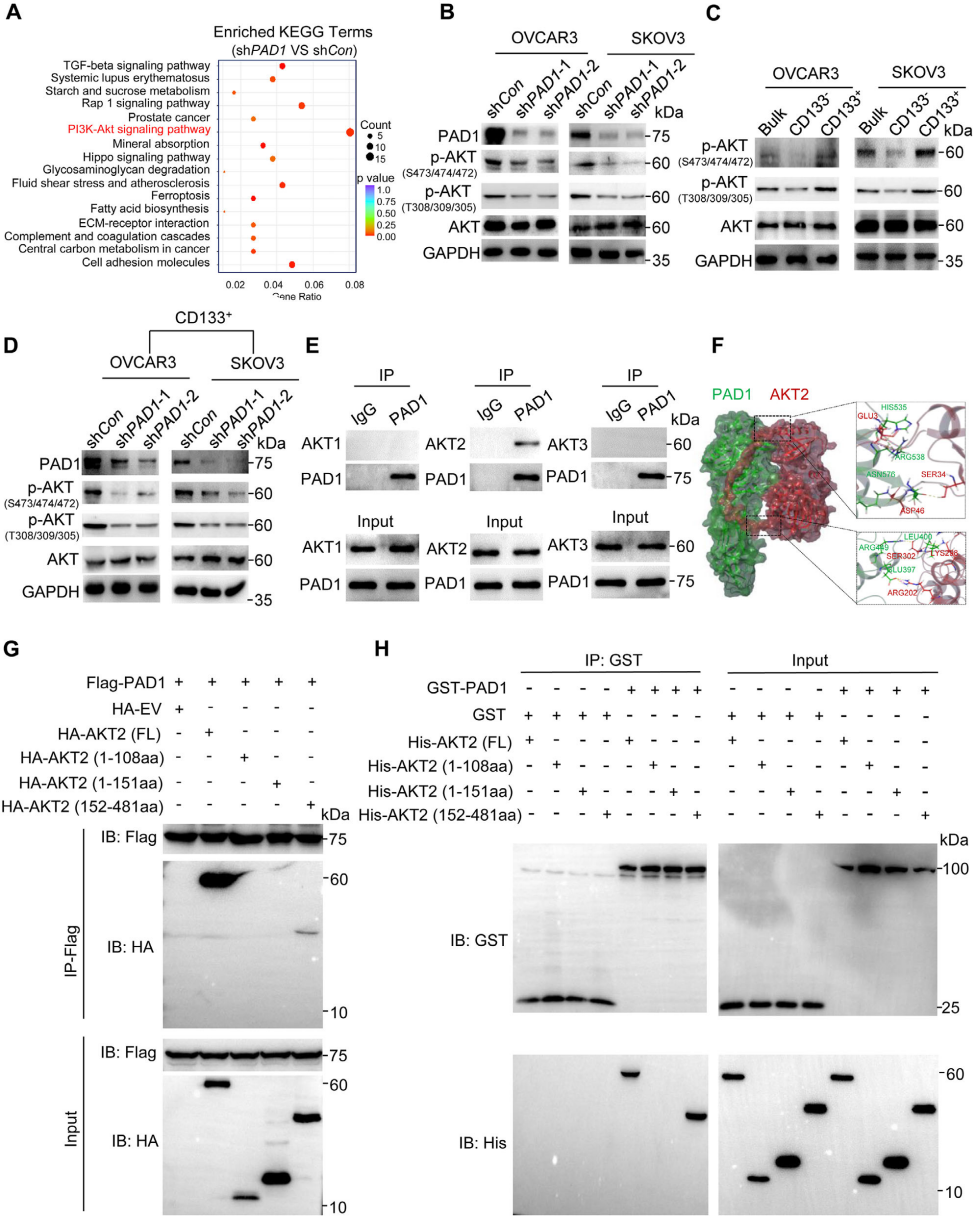
PAD1 maintains the stemness of OCSLCs through activation of AKT signaling. A) The bubble plot showing the enriched signaling pathways of differentially expressed genes upon PAD1 knockdown in OVCAR3 cells. The PI3K‐AKT signaling pathway is highlighted in red. B) Western blot analysis of PAD1, p‐AKT (S473‐AKT1)/(S474‐AKT2)/(S472‐AKT3), p‐AKT (T308‐AKT1)/(T309‐AKT2)/(T305‐AKT3), AKT, and GAPDH in PAD1 knockdown or control OVCAR3 and SKOV3 cells. C) Western blot analysis of p‐AKT (S473‐AKT1)/(S474‐AKT2)/(S472‐AKT3), p‐AKT (T308‐AKT1)/(T309‐AKT2)/(T305‐AKT3), AKT and GAPDH expression in CD133^−^ or CD133^+^ OVCAR3 and SKOV3 cells. D) Western blot analysis of PAD1, p‐AKT (S473‐AKT1)/(S474‐AKT2)/(S472‐AKT3), p‐AKT (T308‐AKT1)/(T309‐AKT2)/(T305‐AKT3), AKT and GAPDH expression in CD133^+^ OVCAR3 and SKOV3 cells upon PAD1 knockdown. GAPDH served as the loading control. E) Co‐immunoprecipitation analysis of the interaction between PAD1 and AKT family proteins in OVCAR3 cells. F) Molecular docking model for the interaction of PAD1 (green) with AKT2 (red). A close‐up view showing the interfacial regions between PAD1 and AKT2. G) Co‐immunoprecipitation analysis of the interaction between Flag‐tagged PAD1 and HA‐tagged AKT2, HA‐tagged AKT2 (1–108 aa), HA‐tagged AKT2 (1–151 aa), HA‐tagged AKT2 (152–481 aa) in HEK293 cells. H) The interaction between PAD1 and full‐length AKT2, as well as AKT2 truncated variants (1‐108 aa, 1–151 aa, and 152–481 aa), was analyzed using a GST pull‐down assay. Recombinant human PAD1 and full‐length AKT2, along with the various AKT2 truncated variants, were incubated with GST‐PAD1 or His‐tagged full‐length AKT2 and its truncated variants.

To elucidate the molecular mechanisms underlying PAD1‐promoted the tumor‐initiating ability of OC cells, we performed RNA sequencing analysis (RNA‐seq) on PAD1 knockdown and parental OVCAR3 (control) cells. Using a twofold expression change threshold and a statistical significance of *p* < 0.05, we identified 350 downregulated and 203 upregulated genes in PAD1 knockdown cells compared to the corresponding expression in control cells (Figure S3A, Supporting Information). An enriched KEGG analysis on these genes identified HALLMARK AKT pathway as the top enriched gene signature (**Figure** **3**A). Consequently, we confirmed that treating OVCAR3 cells with LY294002, an inhibitor known to prevent AKT phosphorylation, led to a global suppression of AKT phosphorylation and dose‐dependent inhibition of the expression of stemness‐related markers (Figure S3B, Supporting Information). Given that AKT activity is aberrantly elevated in the majority of human cancers,^[^ ^23^ ^]^ and that PI3K/AKT signaling is critical in maintaining OCSLC stemness and drug resistance,^[^ ^14^, ^15^ ^]^ we then questioned whether PAD1 also sustained the stemness of OCSLCs through AKT signaling. We observed a significant inhibition of AKT phosphorylation after depleting PAD1 in both OC cell lines (Figure 3B). Note: we analyzed AKT phosphorylation levels using two phospho‐specific antibodies targeting functionally distinct phosphorylation sites: anti‐pAKT(S473/S474/S472) and anti‐p‐AKT(T308/T309/T305). The anti‐p‐AKT(S473/S474/S472) antibody detects endogenous levels of AKT1 only when phosphorylated at S473. This antibody also recognizes AKT2 and AKT3 when phosphorylated at the corresponding residues (S474 in AKT2, S472 in AKT3). Similarly, the anti‐p‐AKT(T308/T309/T305) antibody detects phosphorylated AKT1, AKT2, and AKT3 at their respective homologous residues (T308 in AKT1, T309 in AKT2, and T305 in AKT3). We then observed a marked increase in AKT phosphorylation levels in CD133^+^ OC cells with high PAD1 expression, compared to that of CD133^−^ cells with low PAD1 expression (Figures 3C and 2E). Subsequent depletion of PAD1 in CD133^+^ OC cells led to notable inhibition of AKT phosphorylation (Figure 3D). Given that p‐AKT (S473/S474/S472) is widely recognized as a key biomarker for the complete activation of the PI3K/AKT pathway in tumor research, unless otherwise stated, this antibody was employed throughout the study to assess AKT phosphorylation levels (p‐AKT).

However, the mechanism by which PAD1 regulates AKT activation in OCSLCs remains unclear. To answer this question, we first determined know the localization pattern of PAD1 in OC cells because the subcellular localization of a protein is intrinsically linked to its function. Our cellular fractionation assay clearly demonstrated that endogenous PAD1 was predominantly localized in the cytoplasm of OVCAR3 cells (Figure S3C, Supporting Information), and the overexpression of Flag‐tagged PAD1 also exhibited a cytoplasmic localization pattern (Figure S3D, Supporting Information). Given that we previously demonstrated that PAD2, another PAD family member, can target MEK1 for citrullination and facilitate MEK1 activity in endometrial cancer cells,^[^ ^24^ ^]^ we were prompted to investigate whether PAD1 exerts a similar effect on AKT family members in OC cells. To test this, we first performed co‐immunoprecipitation analysis in OVCAR3 cells using an anti‐PAD1 antibody. AKT kinases include three closely related family members, designated AKT1, AKT2, AKT3.^[^ ^25^ ^]^ Interestingly, only AKT2, and not any other family member, formed a complex with PAD1 (Figure 3E). To validate this interaction in an unbiased manner under endogenous conditions, we performed co‐immunoprecipitation in OVCAR3 cells using an anti‐PAD1 antibody, followed by LC‐MS/MS analysis. AKT2 was successfully identified among the proteins associated with PAD1 (Figure S3E,F, Supporting Information). Moreover, molecular docking studies using ZDOCK calculations also demonstrated strong binding affinity between PAD1 and AKT2 (Figure 3F, Figure S3G, Supporting Information). To further confirm these observations, we transiently transfected Flag‐tagged PAD1 and HA‐tagged AKT2 into HEK293 cells and immunoprecipitated them using an anti‐Flag antibody. A substantial amount of AKT2 co‐precipitated with PAD1. Reciprocally, PAD1 was detected in the material that co‐precipitated with the anti‐HA antibody (Figure S3H, Supporting Information). AKT2 comprises three distinct domains: PH, helix, and kinase domains (Figure S3I, Supporting Information). To identify the AKT2 domains required for the interaction with PAD1, we overexpressed HA‐tagged truncated forms of AKT2 in HEK293 cells together with Flag‐tagged PAD1, followed by immunoprecipitation with an anti‐Flag antibody. The results showed that only the kinase domain of AKT2 bound to PAD1 (Figure 3G). Reciprocally, PAD1 was subsequently co‐precipitated from cells co‐expressed PAD1 and the kinase domain of AKT2 (Figure S3J, Supporting Information). Note: The full‐length AKT2 was used as a positive control. To further verify a direct interaction between PAD1 and AKT2 in vitro, we performed a glutathione S‐transferase (GST) pull‐down assay. Purified His‐tagged full‐length AKT2 protein and its truncated forms were incubated with GST‐tagged PAD1, or with GST alone. The results showed that both His‐AKT2 (full length) and His‐AKT2 (kinase domain) were pulled down by GST‐PAD1, but not by GST alone (Figure 3H), indicating that PAD1 directly binds to the kinase domain of AKT2. Together, these findings strongly suggest that PAD1 is involved in modulating AKT2 kinase activity by directly interacting with its kinase domain.

### 2.4 PAD1‐Catalyzed AKT2 Citrullination Facilitates OCSLC Stemness Maintenance

**Figure 4:**
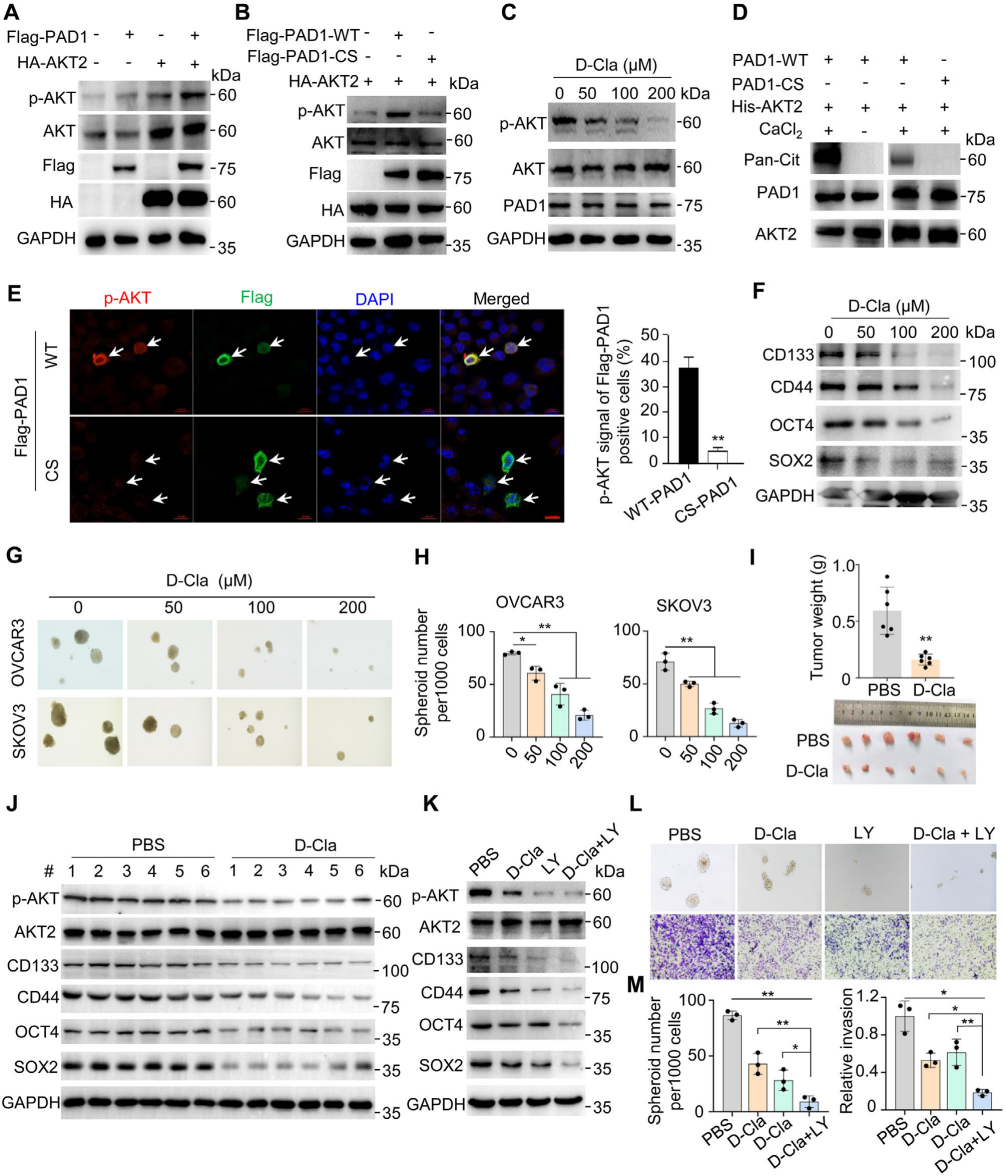
PAD1‐catalyzed citrullination of AKT2 facilitates OCSLC stemness maintenance A) Western blot analysis of p‐AKT (S473/S474/S472), AKT, Flag‐PAD1, HA‐AKT2 and GAPDH in HEK293 cells overexpressed individual Flag‐tagged PAD1, HA‐tagged AKT2, or both. B) Western blot analysis of p‐AKT (S473/S474/S472), AKT, PAD1 (Flag), AKT2 (HA) and GAPDH in HEK293 cells overexpressed AKT2 together with Flag‐tagged PAD1 (Wild Type or catalytic inactive CS mutant). C) Western blot analysis of OVCAR3 cells treated with D‐Cl‐amidine using anti‐p‐AKT (S473/S474/S472), anti‐AKT and anti‐PAD1 antibodies. GAPDH served as loading control. D) Citrullination of His‐tagged AKT2 by PAD1 in vitro in the presence or absence of Ca^2+^, or treated with wild type or catalytic inactive mutant PAD1. The reactions were assessed by western blot using anti‐Pan‐Cit, anti‐PAD1 and anti‐AKT2 antibody. E) Representative immunofluorescence images (left) and quantification (right) of co‐localization of PAD1 and p‐AKT (S473/S474/S472) in OVCAR3 cells overexpressing Flag‐PAD1 (WT or CS). Arrows indicating PAD1‐positive cells. Bar = 10 µm. F) Western blot analysis of CD133, CD44, OCT4, and SOX2 in OVCAR3 cells treated with D‐Cla at indicated dose for 48h. GAPDH served as the loading control. G,H) Representative images of spheroids derived from OVCAR3 or SKOV3 cells treated with D‐Cla at indicated dose (G); The number of spheres with diameters greater than 100 µm was counted (H). I) Dissected tumors collected from nude mice (n = 6) injected with OVCAR3 cells after treatment with D‐Cl‐amidine or PBS (bottom). The upper panel shows the weight of tumors when collected. J) Protein levels of p‐AKT (S473/S474/S472), AKT2, CD133, CD44, OCT4, and SOX2 in tumor lysates. GAPDH served as loading control. K) OVCAR3 cells were treated with 100 µM D‐Cla, 10 µM LY294002 (LY) or a combination of D‐Cla and LY for 48 h, followed by western blot analysis to detect the protein levels of p‐AKT (S473/S474/S472), AKT2, CD133, CD44, OCT4, and SOX2. GAPDH was used as a loading control. L,M) Representative images (L) and quantification (M) for OVCAR3‐derived spheroid with diameters greater than 100 µm cultured in stem cell culture medium in flat bottom ultra‐low attachment plates without FBS (top) and transwell assay (bottom) upon treatment with 100 µM D‐Cla, 10 µM LY294002 (LY) or a combination of D‐Cla and LY294002. Results are presented as mean ± SD, n = 3. *p <0.05, **p < 0.01. E, F: Student's *t*‐test. H, M: one‐way ANOVA.

Previous studies by our group and others demonstrated that PAD‐catalyzed citrullination determines the role of PADs in various cancer cells.^[^ ^24^, ^26^, ^27^ ^]^ To test whether PAD1 may target and regulate AKT2 activity, we first overexpressed AKT2 in the presence or absence of PAD1 and compared the activation state of AKT2. Upon co‐expression of PAD1 and AKT2, AKT2 phosphorylation was activated (**Figure** **4**A), whereas overexpression of the catalytically inactive form of PAD1, carrying a Cys 645 to Ser 645 (CS) mutation, failed to activate AKT (Figure 4B). We then inhibited PAD1 activity using D‐Cl‐amidine (D‐Cla), a newly developed PAD1 inhibitor,^[^ ^28^ ^]^ and found that inhibiting PAD1 enzymatic activity inhibited AKT2 phosphorylation in a dose‐dependent manner (Figure 4C). To further confirm that PAD1 catalyzes AKT2 citrullination, we treated purified His‐tagged AKT2 with recombinant PAD1. The resolved proteins were subsequently subjected to immunoblotting using an anti‐Pan‐Cit antibody, that specifically recognizes citrullinated proteins. The antibody recognized a protein band at 60 kDa from AKT2 purification and did not react with proteins without Ca^2+^ treatment, or those treated with CS PAD1 (Figure 4D). We also overexpressed Flag‐tagged wild‐type (WT) or CS PAD1 in OVCAR3 cells, followed by immunofluorescence analysis to assess the activation status of AKT2. Results showed that in cells overexpressing WT PAD1, AKT2 phosphorylation levels were above the basal level, but not in cells overexpressing CS PAD1 (Figure 4E, Figure S4A, Supporting Information). Collectively, these findings support the hypothesis that the phosphorylation of AKT2 relies on the enzymatic activity of PAD1.

We then investigated whether the role of PAD1 in maintaining OCSLC stemness was intrinsically linked to its enzymatic activity. To test this hypothesis, we first treated OVCAR3 cells with varying concentrations of D‐Cla. Inhibition of PAD1 led to decreases in the expression of cancer stem cell markers (Figure 4F) and tumor spheroid formation ability in both OVCAR3 and SKOV3 cells (Figure 4G,H). Moreover, D‐Cla treatment significantly inhibited tumor growth (Figure 4I), AKT2 activity, and expression of stemness‐related markers (Figure 4J) in individuals from our in vivo tumor xenograft mouse model. Thus, PAD1‐catalyzed AKT2 citrullination seems to be required for maintaining OCSLC stemness. Typically, the combination of different inhibitors targeting the same signaling pathway exhibits stronger synergistic effects than using a single inhibitor. Therefore, we treated OVCAR3 cells with a PAD1 inhibitor in combination with a PI3K/AKT signaling inhibitor. This combined treatment showed an additive inhibitory effect on the expression of stemness‐related markers (Figure 4K), tumorsphere formation and tumor invasion (Figure 4L,M), compared to the effects of individual treatments. Together, it appears that PAD1‐catalyzed citrullination of AKT2 may enhance the activation of AKT signaling pathway, leading to an increase in the expression of OC stemness‐related markers, thereby facilitating the maintenance of OCSLC stemness.

### 2.5 Citrullination of AKT2 at R202 by PAD1 Promotes AKT2 Kinase Activity in OC Cells

**Figure 5:**
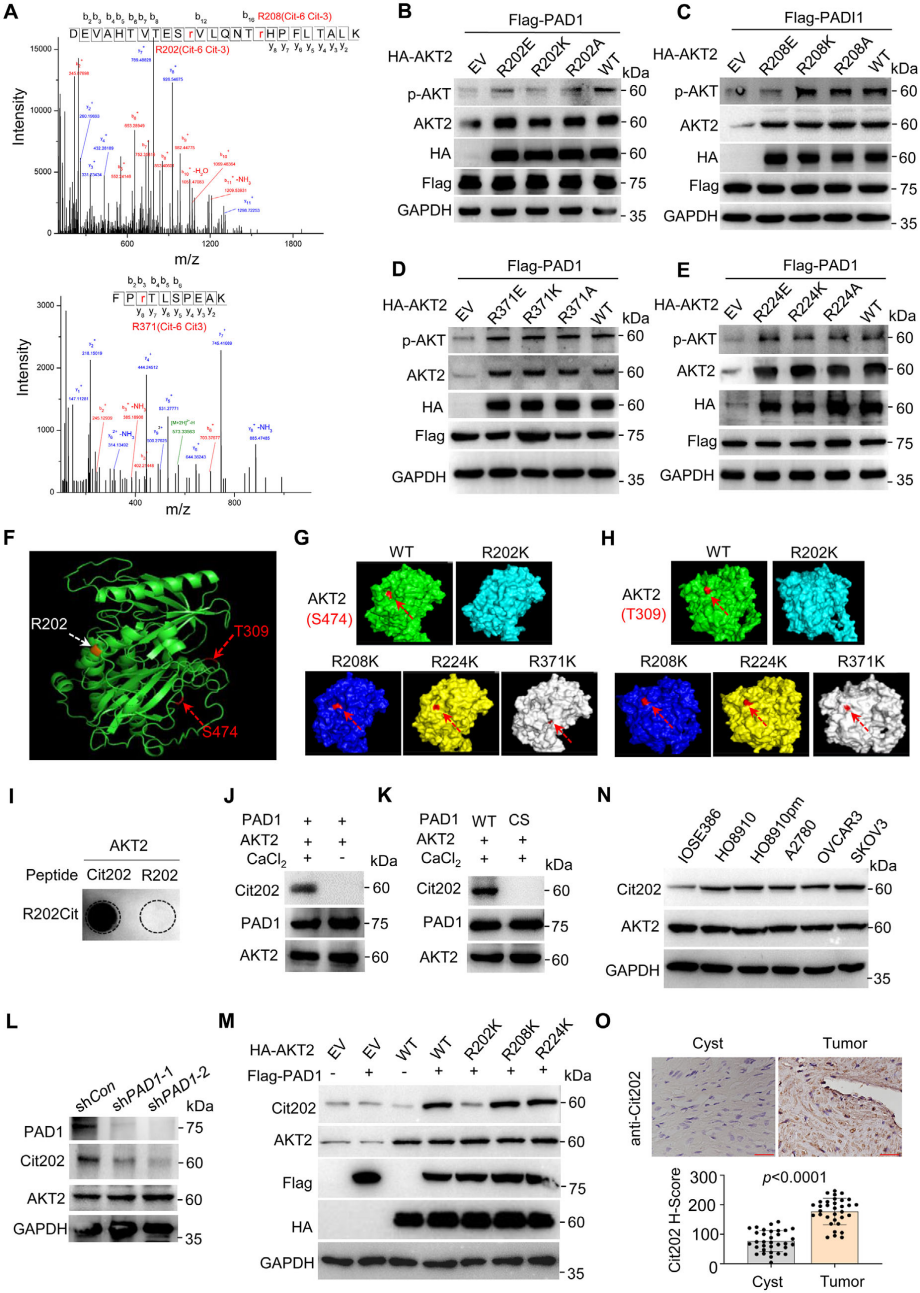
Citrullination of AKT2 at R202 by PAD1 promotes AKT2 kinase activity in OC cells A) MS/MS analysis identifies the citrullination of AKT2 at R202, R208 and R371 sites. Plot shows the fragment spectrum of peptide segment DEVAHTVTESrVLQNTrHPFLTALK (top) and FPrTLSPEAK (bottom). B–E) Western blot analysis of p‐AKT (S473/S474/S472) in HEK293 cells overexpressing AKT2 R/E, R/K, or R/A mutant at R202 (B), R208 (C), R371 (D), R224 (E), together with Flag‐PAD1. GAPDH served as the loading control. F) The schematic diagram of AKT2 protein structure displayed by Pymol, with arrows indicating the positions of R202 (white), T309 and S474 sites (red). G, H) The schematic diagram of AKT2 protein structure displayed by Pymol, with red arrows indicating the exposure of S474 (G) and T309 (H) in WT‐AKT2, R202K‐AKT2, R228K‐AKT2, R371K‐AKT2, and R224K‐AKT2. I) Dot blot analysis of anti‐AKT2 Cit202 in detection of synthetic peptide AKT2 Cit202 and R202. J,K) Citrullination of AKT2 by PAD1 in vitro in the presence or absence of Ca^2+^ (J), or treated with wild type or catalytic inactive PAD1 (K). The reactions were assessed by western blot using anti‐AKT2 Cit202, anti‐PAD1 and anti‐AKT2 antibodies. L) Western blot analysis of AKT2 Cit202 upon PAD1 depletion in OVCAR3 cells. GAPDH served as the loading control. M) Western blot analysis of Cit202 of AKT2 in HEK293 cells overexpressed AKT2 R202K R208K, and R224K, together with Flag‐PAD1. N) Western blot analysis of AKT2 Cit202 in normal ovarian epithelial cell lines IOSE386 and different ovarian cancer cell lines. GAPDH served as the loading control. O) Top: Immunohistochemistry analysis of Cit202 in ovarian cancer and adjacent tissues of clinical patients, original magnification. Bottom: Statistical analysis of H‐Score (histochemical score) was conducted to evaluate Cit202 expression levels in both ovarian cancer and cyst tissues. Bar = 20 µm. O: Student's *t*‐test.

To identify those AKT2 arginine (R) residues that may undergo citrullination by PAD1, we purified His‐tagged AKT2 proteins previously and treated with PAD1. The protein band corresponding to the mass of citrullinated AKT2 was excised from the gel and subjected to mass spectrometry (MS) analysis. LC‐MS/MS analysis revealed that PAD1 preferentially citrullinated the arginine (R) residues at R202, R208, and R371 (**Figure** **5**A), which are located within the kinase domain of AKT2 (Figure S3I, Supporting Information). As serine 474 (S474) phosphorylation is essential for maximal AKT2 kinase activity,^[^ ^29^ ^]^ we wondered whether AKT2 citrullination at R202/R208/R371 affected phosphorylation at S474. To test this hypothesis, we generated R→E, R→K, and R→A mutant of AKT2 on R site separately, and transfected them into HEK293 cells to test their activity in the presence of PAD1 (Figure S4B, Supporting Information). Note: Given that citrullination neutralizes positively charged arginine residues, R→E, R→A and R→K mutants were generated to exclude the possibility that charges of these residues play the roles. Results showed that overexpression of WT AKT2 increased AKT2 phosphorylation levels, and mutation of R202 on AKT2 dramatically decreased AKT2 phosphorylation (Figure 5B). However, mutation of R208 (Figure 5C) or R371 (Figure 5D) did not affect AKT2 activation. Of note, R208 R→K and R→A mutants did not affect p‐AKT2 activation, whereas R→E mutant still retained its inhibitory effect (Figure 5C). These results indicated that the presence of a positive charge or no charge on the R208 residue may be essential for maintaining the kinase activity of AKT2. However, the introduction of a negative charge could potentially alter the protein's spatial conformation, which may in turn hinder the activation of AKT2. Thus, the decrease in p‐AKT2 levels caused by AKT2 R208E mutant can be attributed to charge alterations rather than citrullination at R208. A mutation at R224 was chosen as the negative control, as it was not identified as a potential site for citrullination by the LC‐MS/MS analysis. Co‐transfection of mutated form of R224 with PAD1 could not inhibit AKT2 phosphorylation (Figure 5E). These results suggested that R202 is the target site for PAD1 to citrullinate on AKT2, which is a critical for AKT2 kinase activity. Activation of AKT2 involves phosphorylation of T309 and S474.^[^ ^30^ ^]^ To better visualize the spatial structure and relationship between R202 and these two primary phosphorylation sites in AKT2, the secondary structure of the AKT2 protein structure was modeled using information from the Protein Data Bank, where R202 (white arrow), T309 and S474 (red arrows) sites are denoted (Figure 5F). Wild‐type and mutant AKT2 protein structure models were constructed and visualized using I‐TASSER and PyMol software (The PyMOL Molecular Graphics System, version 2.0, Schrödinger, LLC). Interestingly, only the R202 mutation disrupted the exposure of S474 (Figure 5G) and T309 (Figure 5H) on AKT2 protein surface, while mutations at other R sites had little effect (Figure 5G,H). This analysis may help explain why the R202 mutant specifically prevents AKT2 phosphorylation, but not the other mutants. We then utilized a specific anti‐p‐AKT2(S474) monoclonal antibody that recognizes endogenous AKT2 protein only when phosphorylated at S474, and confirmed a decrease of AKT2 phosphorylation level at S474 following PAD1 knockdown. Notably, PAD1 knockdown did not alter the phosphorylation level of AKT1 at S473 as detected by the anti‐p‐AKT1(S473) mAb (Figure S4C, Supporting Information); furthermore, the co‐overexpression of AKT2 and wild‐type PAD1 induced the activation of p‐AKT2(S474), while AKT1 phosphorylation at S473 was not affected by PAD1 overexpression. As a control, AKT1 phosphorylation did not seem to be affected under AKT2 R202 mutation in the presence of PAD1 (Figure S4D, Supporting Information).

To examine the in vivo citrullination of AKT2 at R202, we raised an antibody that recognizes citrullinated AKT2 using a peptide corresponding to amino acids 193 to 207 (Figure 5I, Figure S4E, Supporting Information). Antibody titers and specificity were verified by dot blotting using a synthetic citrullinated AKT2 peptide (Cit202: EVAHTVTES‐Cit‐VLQNT) and a non‐citrullinated peptide mimicking modified AKT2 (Arg202: EVAHTVTES‐R‐VLQNT) (Figure S4E, Supporting Information). Notably, AKT2 R202 is highly conserved across multiple species (Figure S4F, Supporting Information). The anti‐Cit202 antibody clearly demonstrated the citrullination of AKT2, showing the same molecular weight as that of anti‐MC antibody from purified AKT2 treated with PAD1, and was not reactive with proteins without Ca^2+^ treatment (Figure 5J) or treated with catalytically inactive PAD1 (Figure 5K). In addition, the antibody also effectively detected the signal from OVCAR3 control cells, and this signal decreased when PAD1 was depleted (Figure 5L), indicating that the antibody specifically recognized the citrullination of R202 in a PAD1‐dependent manner. To avoid potential cross‐reactivity with other arginine sites in AKT2, we also tested this antibody on HEK293 cells overexpressing AKT2 WT or mutants in the presence of PAD1. It was observed that mutation of R208K and R224K did not affect the ability of anti‐Cit202 antibody to recognize R202 citrullination in AKT2. However, mutation of R202K almost completely eliminated this signal (Figure 5M), further confirming the specificity of the antibody. Next, the citrullination levels of AKT2 R202 were obviously higher in a range of OC cell lines than that in IOSE386 control cells (Figure 5N). Importantly, this antibody is applicable for immunohistochemistry analysis of clinical ovarian tumor tissues. The results showed that Cit202 staining was strongly positive in OC tissue sections, while less positive or nearly negative in the ovarian cyst sections (Figure 5O). Together, these findings strongly suggested that citrullination of AKT2 at R202 by PAD1 may promote AKT2 kinase activity in OC cells.

### 2.6 Overexpression of AKT2 R202K in OC Cells Impedes the Maintenance of OCSLC Stemness

**Figure 6:**
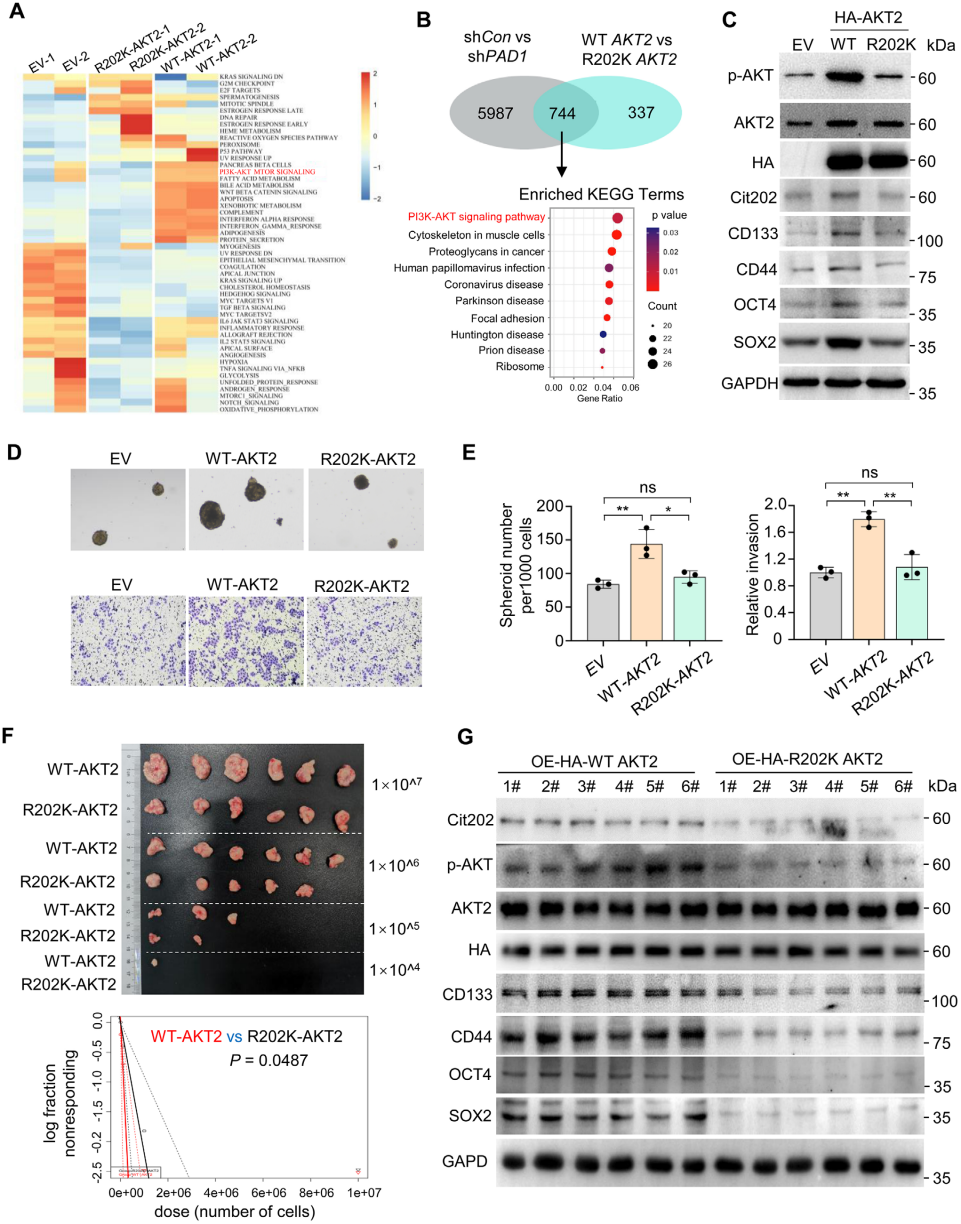
Overexpression of AKT2 R202K in OC cells impedes the maintenance of OCSLC stemness A) KEGG analysis of the enriched signaling pathways from differentially expressed genes between OVCAR3 cells overexpressing WT‐AKT2 and that of R202K‐AKT2. The PI3K‐AKT signaling pathway is highlighted in red. B) The Venn diagram shows the overlapping transcripts that were altered in both AKT2 R202K overexpression cells and those changed in PAD1 KD OVCAR3 cells (top); The gene enrichment analysis identifies PI3K‐AKT pathway (in red) as the enriched gene signature (bottom). C) Western blot analysis of p‐AKT (S473/S474/S472), AKT2, Cit202, CD133, CD44, OCT4, SOX2 and GAPDH in OVCAR3 cells overexpressed HA‐AKT2 (WT or R202K). EV represents the empty vector control. D) Representative images of spheroids derived from OVCAR3 cells overexpressed HA‐AKT2 WT or R202K mutant (top), and cells in transwell assay (bottom). EV represents the empty vector. E) Quantification of the spheroids with diameters greater than 100 µm (D, top), and the relative invasion ability of OVCAR3 cells (D, bottom). F) Top: Dissected tumors collected from nude mice in extreme limiting dilution analysis (n = 6, for each cell injection group). Mice injected with OVCAR3 cells overexpressed HA‐WT‐AKT2 on the left flank and HA‐R202K‐AKT2 on the right flank. Bottom: The OCSLC frequency analysis for the OVCAR3 cells overexpressed HA‐WT‐AKT2 versus OVCAR3 cells overexpressed HA‐R202K‐AKT2 was calculated by ELDA software. A plot of the log fraction of nonresponding versus the number of cells, the slope of the line representing the estimated log‐OCSLC fraction and the dotted lines give the 95% confidence interval. G) Western blot analysis of Cit202, p‐AKT (AKT1 S473/AKT2 S474/AKT3 S472), AKT2, CD133, CD44, OCT4, and SOX2 in tumor tissue collected from (F). GAPDH served as loading control. Results are presented as mean ± SD, n = 3. *p <0.05, **p < 0.01. E: one‐way ANOVA.

We next generated stable OVCAR3 cells overexpressing HA‐tagged WT AKT2, HA‐tagged AKT2 R202K, or the empty vector as control, followed by the RNA‐seq of these cells. Gene Set Variation Analysis (GSVA) disclosed an enriched PI3K‐AKT pathway in WT‐AKT2 overexpressed cells, while R202K‐AKT2 overexpression diminished such an enrichment (**Figure** **6**A). Interestingly, a comparison of the transcripts that were altered in R202K‐overexpressing cells with those changed in PAD1 KD OC cells showed a high degree of similarity between the two sets (Figure 6B). Moreover, gene enrichment analysis of the 744 overlapped genes again revealed PI3K‐AKT pathway as the enriched gene signature (Figure 6B). We then confirmed that the R202K mutant OVCAR3 cells exhibited reduced AKT2 phosphorylation and decreased R202 citrullination compared to the WT‐AKT2 overexpressed cells, similar to the extent observed in EV cells (Figure 6C). Interestingly, R202K mutant also resulted in a decreased expression of the cancer stemness‐related markers, compared to that of WT AKT2 overexpressing cells (Figure 6C). Such effects were also observed in S474A mutant cells but not in R208K mutant (used as a control) (Figure S5A, Supporting Information). To further test whether PAD1‐catalyzed AKT2 citrullination modulates the stemness of OC cell, the tumorsphere formation and transwell invasion assays were monitored (Figure 6D). The R202K mutant substantially decreased the OC cell spheroid formation and invasion (Figure 6E), indicating that AKT2 R202 citrullination is exclusively essential for the stemness maintenance of OC cells. To further confirm the critical role of R202 citrullination in maintaining the stemness of OC cells, we generated sgRNA‐mediated PAD1 knockout cells (a heterogeneous cell population), followed by a rescue strategy. Co‐overexpression of AKT2 and PAD1 in PAD1 knockout cells demonstrated that only wild‐type PAD1 was capable of restoring the expression of tumor stem cell‐related genes, accompanied by an increase in the levels of AKT2 citrullination and phosphorylation; in contrast, catalytically inactive PAD1 failed to elicit these changes (Figure S5B, Supporting Information). As a control, co‐expression of WT‐PAD1 and AKT2 R202K mutant in PAD1 knockout cells failed to restore the above changes (Figure S5C, Supporting Information). Consequently, only the co‐expression of WT‐PAD1 and WT‐AKT2 could maintain both the tumorsphere formation efficiency and invasion of OC cells, while neither the AKT2 R202K mutant nor CS PAD1 exhibited these abilities (Figure S5D,E, Supporting Information). The intraperitoneal metastatic xenograft mouse model further demonstrated that animals injected with OC cells overexpressing R202K‐AKT2 exhibited significantly fewer metastatic implants and a reduced ascites volume compared to those injected with WT‐AKT2 overexpressing cells (Figure S5F,G, Supporting Information). Notably, PAD1‐mediated citrullination of AKT2 appears to specifically contribute to maintaining the stemness of OC cells compared to other AKT family members, as neither the rescue of AKT1 nor AKT3 induced a similar effect (Figure S6, Supporting Information). Additionally, ELDA performed in nude mice inoculated with OVCAR3 cells overexpressed WT AKT2 or AKT2 R202K confirmed that AKT2 R202K overexpression reduces OCSLC frequency (Figure 6F). Again, the levels of R202 citrullination, AKT2 phosphorylation, and expression of cancer stemness‐related markers were all found to be decreased in the tumors from the mice inoculated with the AKT2 R202K overexpressed cells (Figure 6G). These results suggested that the R202K mutation in OC cells impedes the maintenance of OCSLC stemness.

### 2.7 CEBPβ May Serve as a Crucial Downstream Regulatory Target of PAD1‐AKT2 Signaling

**Figure 7:**
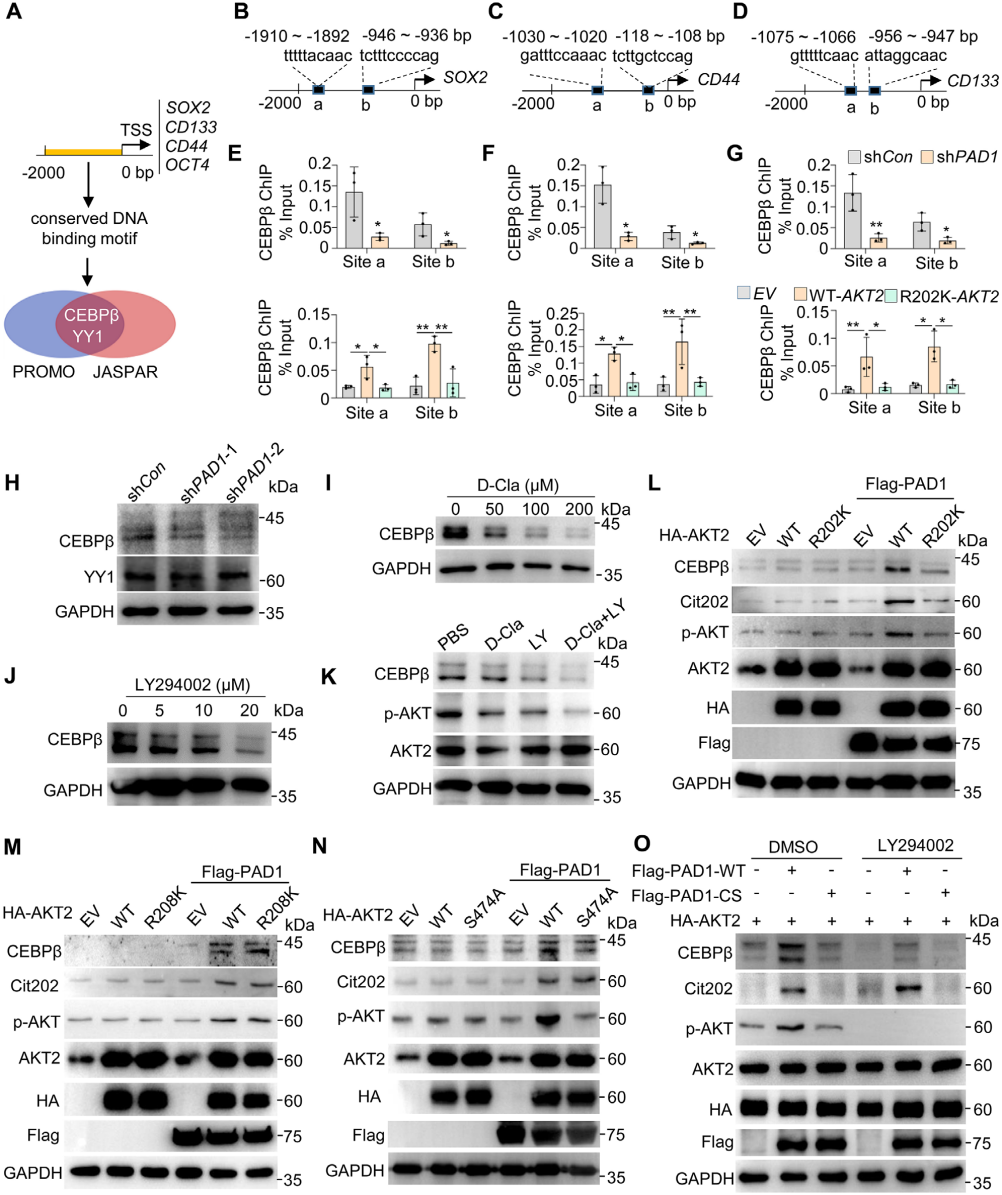
CEBPβ may serve as a crucial downstream regulatory target of PAD1‐AKT2 signaling A) Schematic diagram showing the prediction of common transcription factors for tumor stemness‐related genes through PROMO and JASPAR websites. B–D) The schematic diagram indicates the locations of the CEBPβ binding motifs relative to the TSS for *SOX2* (B), *CD44* (C), and *CD133* (D) used for ChIP assay. E–G) ChIP‐qPCR analysis of CEBPβ binding at *SOX2* (E), *CD44* (F), and *CD133* (G) promoters in OVCAR3 cells. H) Western blot analysis of CEBPβ and YY1 upon PAD1 depletion in OVCAR3 cells. GAPDH served as the loading control. I, J) Western blot analysis of CEBPβ in OVCAR3 cells treated with a range of D‐Cla (I) or LY294002 (J) at indicated dose for 48 h. GAPDH served as the loading control. K) Western blot analysis of CEBPβ, p‐AKT (S473/S474/S472), and AKT2 in OVCAR3 cells treated with 100 µM D‐Cla, 10 µM LY294002 or a combination of D‐Cla and LY294002 for 48 h. GAPDH served as the loading control. L–N) Western blot analysis of CEBPβ, Cit202, p‐AKT (S473/S474/S472), HA‐AKT2, and Flag‐PAD1 in HEK293 cells overexpressed HA‐AKT2 R202K (L), R208K (M), S474A (N) or a combination of HA‐AKT2 with Flag‐PAD1. GAPDH served as the loading control. O) Western blot analysis of CEBPβ, Cit202, p‐AKT (S473/S474/S472), HA‐AKT2, and Flag‐PAD1 in HEK293 cells overexpressed with AKT2 and Flag‐PAD1 (WT or CS) upon LY294002 treatment, compared to DMSO treatment. GAPDH used as loading control. Results are presented as mean ± SD, n = 3. *p < 0.05, **p < 0.01. E, F, G top: Student's *t*‐test; bottom: one‐way ANOVA.

To further understand how the PAD1/AKT signaling pathway regulates the expression of tumor stemness‐related genes, we used the PROMO and JASPAR databases to predict the transcription factor‐binging sites in the proximal promoters of *CD133*, *CD44*, *SOX2*, and *OCT4* involved in gene regulation. After analyzing the 2000 bp promoter regions of the genes, only two shared transcription factors, CCAAT/Enhancer Binding Protein Beta (CEBPβ) and Yin Yang 1 (YY1), were predicted to be common regulators of all four genes (**Figure** **7**A). To confirm this, we performed chromatin immunoprecipitation (ChIP) coupled with qPCR assays using anti‐CEBPβ (Figure 7B–D) and anti‐YY1 (Figure S7A–C, Supporting Information, top) antibodies on the specific binding sites in OVCAR3 cells. The results showed enriched CEBPβ to the chromatin fragments containing CEBPβ binding sites (sites a and b at each gene promoter) on *SOX2*, *CD44*, and *CD133* promoter regions (Figure S7D, Supporting Information). In contrast, YY1 did not appear to bind to any of these regions (Figure S7A–C, Supporting Information, bottom). Regarding *OCT4*, CEBPβ or YY1 binding to the promoter could not be tested due to the unavailability of suitable primer sets for amplifying the regions containing the corresponding binding motifs. To further verify whether transcription of *SOX2*, *CD44*, and *CD133* was regulated by CEBPβ, the activity of the CEBPβ binding sites in their promoters was examined by luciferase reporter assays. The transcription of luciferase genes controlled by the proximal promoters of *SOX2*, *CD44*, or *CD133* was activated upon CEBPβ overexpression (Figure S7E, Supporting Information). Actually, CEBPβ has also been reported to promote the expression of stemness‐related markers in other cancers and be involved in maintenance of cancer stem‐like cell stemness.^[^ ^31^, ^32^ ^]^ Therefore, these data suggested that CEBPβ may promote the malignant progression of OC by modulating the expression of stemness‐related markers in OC cells.

CEBPβ enrichment to the promoters of the four stemness‐related genes was also R202 citrullination‐dependent, as either depletion of PAD1 or overexpression of AKT2 R202K in OVCAR3 cells prevented CEBPβ occupancy of these sites (Figure 7E–G). Therefore, the transcription levels of these four genes decreased in PAD1 knockdown cells (Figure S7F, Supporting Information) and in OVCAR3 cells overexpressing R202K AKT2 (Figure S7G, Supporting Information). These results suggested that CEBPβ may serve as a crucial regulatory target downstream of the PAD1‐AKT signaling pathway controlling the transcription of cancer stemness‐related markers. To further confirm this hypothesis, we first showed that depletion of PAD1 reduced CEBPβ expression, but not that of YY1 (Figure 7H). We than treated OVCAR3 cells with either D‐Cla (Figure 7I) or LY294002 (Figure 7J), and observed a dose‐dependent decrease in CEBPβ expression under either treatment. The combination of D‐Cla and LY294002 showed an additive inhibitory effect on the expression of CEBPβ, as compared to that of each individual treatment (Figure 7K). Given that citrullination of AKT2 R202 catalyzed by PAD1 may facilitate AKT2 phosphorylation in OC cells (Figure 6), we hypothesized that AKT2 citrullination at R202 would also affect CEBPβ expression, ultimately leading to the activation of stemness‐related markers. Therefore, we continued exploring the effect of AKT2 R202 mutation on CEBPβ expression. The results showed that overexpression of AKT2 R202K in the presence of PAD1 resulted in a significant decrease in the expression of CEBPβ, while no effect was observed when either AKT2 WT or the R208K mutant were used instead (Figure 7L,M), suggesting that CEBPβ expression is also dependent on AKT2 R202 citrullination. Additionally, in the absence of PAD1, neither WT nor any mutant form of AKT2 was able to maintain AKT2 kinase activity or CEBPβ expression, thereby underscoring the essential role of R202 citrullination in facilitating AKT2 phosphorylation and CEBPβ expression (Figure 7L,M). We also noticed that while the overexpression of AKT2 S474A abolished AKT2 phosphorylation and did not enhance CEBPβ expression, partial restoration of CEBPβ could still occur through AKT2 citrullination at R202 in the presence of PAD1 (Figure 7N). This finding suggest that, in addition to the role of AKT2 phosphorylation in activating CEBPβ expression, AKT2 citrullination may also promote downstream CEBPβ expression in an AKT2 phosphorylation‐independent manner. This might be achieved by altering the protein structure or activity of AKT2 to help maintain the active AKT2 site properly structured, thus enhancing its activity on CEBPβ expression. To further confirm this hypothesis, we rescued either WT or the catalytically inactive PAD1 in the presence of AKT2 in HEK293 cells (Figure 7O), and observed an active R202 citrullination and upregulated CEBPβ expression upon WT PAD1 overexpression (lane 2), while the mutant PAD1 did not (lane 3). We then inhibited AKT2 phosphorylation using LY294002 to suppress CEBPβ expression and assessed whether PAD1 could rescue CEBPβ. Notably, the overexpression of WT PAD1 partly restored CEBPβ expression (compare lanes 5 and 6). We also overexpressed Flag‐tagged CEBPβ in control OVCAR3 cells and in cells with PAD1‐knockout (Figure S8A, Supporting Information) or AKT2 knockout (Figure S8B, Supporting Information). The results showed that CEBPβ overexpression upregulated the expression of tumor stemness genes in control cells, however, this effect was abolished in cells with PAD1‐knockout (Figure S8A, Supporting Information) or AKT2 knockout (Figure S8B, Supporting Information). Conversely, transient knockdown of CEBPβ in control OVCAR3 cells led to a decrease in the expression of these tumor stemness genes, while si*CEBPβ* treatment in either PAD1‐knockout (Figure S8C, Supporting Information) or AKT2 knockout (Figure S8D, Supporting Information) cells did not affect the expression of these genes, likely due to the substantial reduction in basal CEBPβ levels following PAD1 or AKT2 depletion. Collectively, these results suggested that PAD1‐catalyzed AKT2 citrullination at R202 facilitates AKT2 phosphorylation, or help AKT2 properly structured, leading to an active form of AKT2 that enhances CEBPβ expression and ultimately promotes the transcription of cancer stemness‐related genes.

### 2.8 Inhibiting PAD1 Resensitizes OVCAR3‐CisR Cells to Cisplatin through Suppression of AKT2/CEBPβ Signaling Pathway

**Figure 8:**
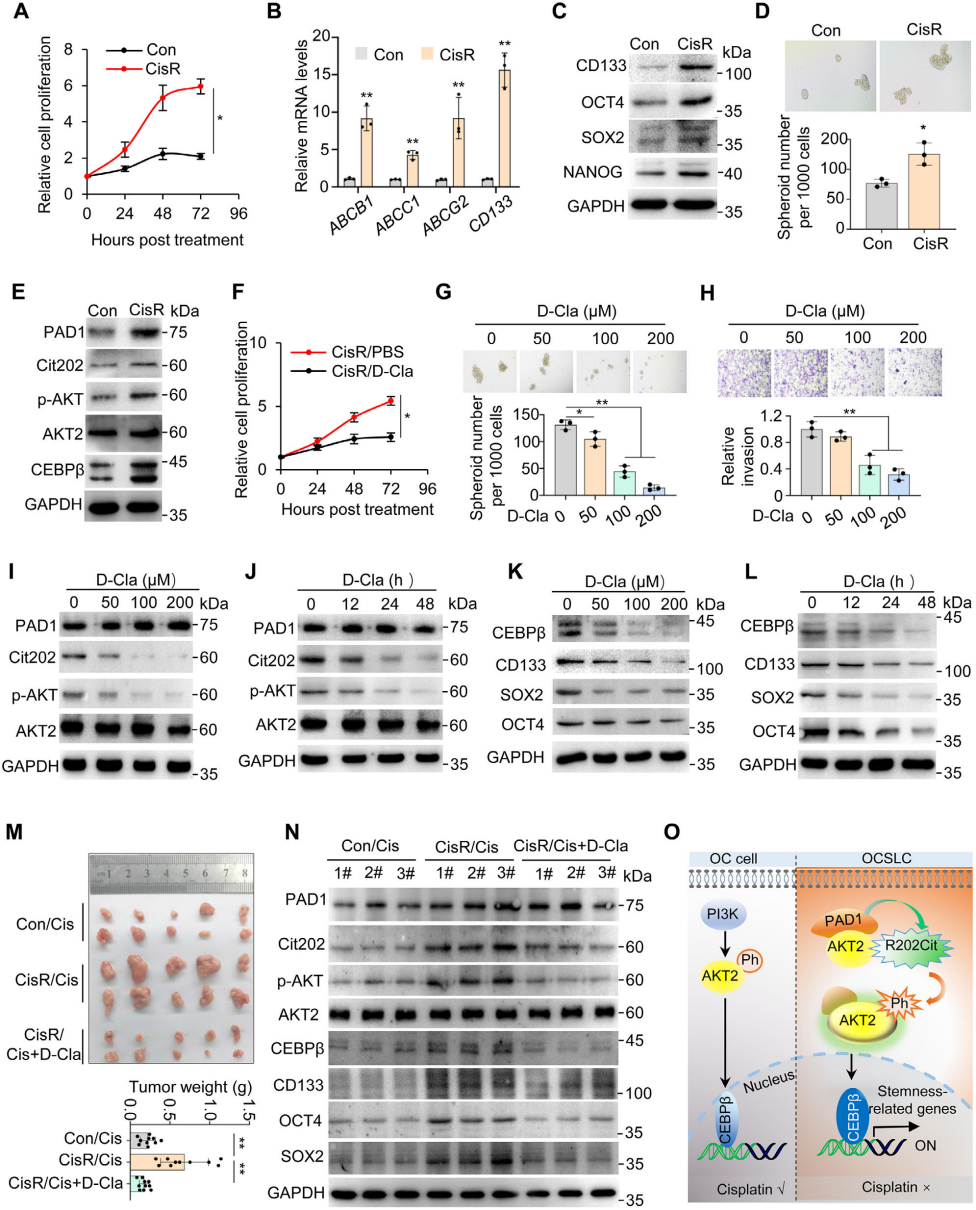
Inhibiting PAD1 resensitizes OVCAR3‐CisR cells to cisplatin through suppression of AKT2/CEBPβ signaling pathway A) Relative cell proliferation with CCK8 assay in OVCAR3 control (Con) and CisR cells under 5 µM Cis treatment. B) Relative mRNA levels of *ABCB1, ABCC1, ABCG2*, and *CD133* were detected in OVCAR3 control and CisR Cells by RT‐qPCR. *GAPDH* was used as the reference control. C) Western blot analysis of CD133, OCT4, SOX2, NANOG in OVCAR3 control and CisR cells. GAPDH served as the loading control. D) Representative images of spheroids derived from OVCAR3 control and CisR cells, with diameters greater than 100 µm cultured in stem cell culture medium in flat bottom ultra‐low attachment plates without FBS (top) and quantification (bottom). E) Western blot analysis of PAD1, Cit202, p‐AKT (S473/S474/S472), AKT2, CEBPβ in OVCAR3 control and CisR cells. GAPDH served as the loading control. F) Relative cell proliferation with CCK8 assay in OVCAR3 CisR cells under 5 µM Cis treatment together with 200 µM D‐Cla or PBS (as a control). G,H) Representative images (top) and quantification (bottom) for OVCAR3 CisR‐derived spheroids with diameters greater than 100 µm cultured in stem cell culture medium in flat bottom ultra‐low attachment plates without FBS (G) and transwell assay (H) under D‐Cla treatment. I,J) Western blot analysis of OVCAR3 CisR cells treated with D‐Cla at indicated dose for 48 hr (I), or 200 µM D‐Cla at indicated time (J) using anti‐PAD1, anti‐Cit202, anti‐p‐AKT (S473/S474/S472), anti‐AKT2. GAPDH served as loading control. K,L) Western blot analysis of OVCAR3 CisR cells treated with D‐Cla at indicated dose for 48 hr (K), or 100 µM D‐Cla at indicated time (L) using anti‐CEBPβ, anti‐CD133, anti‐SOX2, anti‐OCT4. GAPDH served as loading control. M) Dissected tumors collected from nude mice (n = 6) injected with OVCAR3 control cells or OVCAR3 CisR cells after treatment with Cis or co‐administration of D‐Cla with Cis (top). The bottom panel shows the weight of tumors when collected. N) Western blot analysis of PAD1, Cit202, p‐AKT (S473/S474/S472), AKT2, CEBPβ, CD133, OCT4, and SOX2 in tumor tissue collected from (M). GAPDH served as the loading control. O) The proposed model illustrates how AKT2 citrullination at R202, catalyzed by PAD1, activates the AKT signaling pathway, leading to increased CEBPβ expression and enhanced transcription of tumor stemness‐related genes, which in turn raises ovarian cancer malignancy. Results are presented as mean ± SD, n = 3. *p < 0.05, **p < 0.01. A, B, D, F: Student's *t*‐test; G, H, M: one‐way ANOVA.

Chemotherapy resistance is the primary determinant of poor prognosis in patients with advanced OC, and OCSLCs are closely associated with chemotherapy resistance. Given the critical role of PAD1‐mediated AKT activation in OCSLCs, we wondered whether this signaling axis is also implicated in the development of chemotherapy resistance in OC. To test this, we first established a cisplatin‐resistant OVCAR3 cells (CisR) by prolonged exposure of OVCAR3 cells to cisplatin (Cis). The CisR cells eventually survived in the culture medium containing 5 µM Cis (**Figure** **8**A), and exhibited elevated expression levels of genes associated with Cis resistance (Figure 8B, Figure S9A, Supporting Information). Compared with that in OVCAR3‐control (Con) cells, which exhibited lower resistance to Cis, the expression of tumor stemness‐related molecules was clearly upregulated in CisR cells (Figure 8C), and the tumorsphere formation ability of CisR cells was also significantly upregulated (Figure 8D). These findings confirm that the cells resistant to Cis are highly similar to cancer stem‐like cells in OC. As expected, the expression levels of PAD1 and PAD1‐catalyzed citrullination of AKT2 at R202 were found to be elevated, accompanied by increased activation of p‐AKT and enhanced expression of CEBPβ (Figure 8E). The proliferation of OVCAR3‐CisR cells (regularly maintained under 5 µM Cis) treated with D‐Cla was significantly suppressed compared to that of PBS treated cells (Figure 8F). Additionally, inhibiting PAD1 enzymatic activity also significantly attenuated the abilities of tumorsphere formation and tumor cell invasion in CisR cells in a dose‐dependent manner (Figure 8G,H).

To demonstrate that the inhibitory effect of D‐Cla on OVCAR3‐CisR cells also occurs primarily through inhibiting the PAD1/AKT2/CEBPβ signaling axis, we exposed OVCAR3‐CisR cells to D‐Cla at a range of concentrations and durations. Results showed that inhibiting PAD1 enzymatic activity effectively prevented AKT2 citrullination and phosphorylation in a dose‐ and time‐dependent manner (Figure 8I,J). D‐Cla treatment also resulted in dose‐ and time‐dependent decreases in the expression of cancer stem cell markers (Figure 8K,L). In addition, the combined treatment of OVCAR3‐CisR cells with D‐Cla and AZD5363 (an FDA‐approved AKT inhibitor) demonstrated an additive inhibitory effect on the activation of AKT, as well as on the expression levels of CEBPβ and cancer stemness‐related molecules (Figure S9B, Supporting Information). Accordingly, a combined treatment also resulted in a more pronounced reduction in cell proliferation (Figure S9C, Supporting Information), tumorsphere formation, and tumor invasion than in individual treatments (Figure S9D,E, Supporting Information). Furthermore, the in vivo tumor xenograft mouse model confirmed that a single treatment with Cis effectively reduced tumor growth in the nude mice inoculated with OVCAR3‐Con cells, while it failed to inhibit tumor growth in those inoculated with CisR cells. Intriguingly, the co‐administration of D‐Cla and Cis effectively suppressed tumor growth in mice inoculated with CisR cells, reducing the size of the tumors to sizes comparable to those observed in the OVCAR3‐Con group treated with Cis alone, suggesting that D‐Cla treatment may help resensitize CisR cells to Cis (Figure 8M). Noteworthy, the combined treatment effectively inhibited the activation of AKT2 and suppressed the expression of CEBPβ and stemness‐related markers in tumor tissue obtained from nude mice (Figure 8N). These results indicated that the PAD1/AKT2/CEBPβ signaling axis may potentially contribute to the development of Cis resistance in OC, and targeting the citrullination of AKT2 catalyzed by PAD1 is anticipated to be a novel therapeutic strategy for overcoming this resistance.

## 3 Discussion

In clinical practice, OC treatment is challenged by chemoresistance and recurrence.^[^ ^3^, ^4^ ^]^ The presence of CSLCs, a subset of drug‐resistant tumor cells, is representing a crucial driving force behind malignant progression, metastasis, and ultimately tumor recurrence.^[^ ^5^, ^6^, ^9^ ^]^ Therefore, the efforts to further dissect the molecular mechanisms underlying the activation and maintenance of stemness in OCSLCs will help address the issue of chemoresistance and recurrence in OC, and may also provide new insights for identifying more effective and selective therapeutic targets for OC treatment. In this study, we observed an increased expression of PAD1 in OC cells, particularly within the CD133^+^ subset or CisR OC cells, which was positively associated with the upregulation of stemness‐related markers. Depletion of PAD1 or inhibition of PAD1 enzymatic activity reduces the expression of stemness‐related markers, and inhibits the tumor‐initiating ability of OC cells both in vitro and in vivo. Importantly, treatment with a PAD1 inhibitor resensitized CisR OC cells to Cis. Regarding the molecular mechanism by which PAD1 maintains the stemness of OCSLCs, we demonstrated that PAD1‐catalyzed AKT2 citrullination at R202 facilitated the phosphorylation of the protein in OCSLCs and promoted CEBPβ expression, leading to enhanced binding of CEBPβ to the promoters of the cancer stemness‐related genes and, ultimately, to their transactivation (Figure 8O). These data led us to conclude that PAD1 plays a crucial role in driving OC tumorigenesis, primarily by maintaining the stemness of OCSLCs. To date, most studies have focused on the roles of PAD2 and PAD4 in cancer, whereas that of other members of the PAD family has received less attention.^[^ ^26^, ^33^, ^34^ ^]^ The novel function of PAD1 in OC cells, particularly in OCSLCs, further enriches our understanding of the influence of PADs and PADs‐catalyzed citrullination on tumor progression.

It is particularly important to note that PAD1 specifically interacts with and catalyzes the citrullination of AKT2, thereby enhancing its kinase activity in OCSLCs. There are three AKT homologs, AKT1, AKT2, and AKT3, that share a similar structural topology but differ in their tissue distribution, expression patterns, and non‐redundant functions in various tumor types and cancer progression.^[^ ^25^, ^35^, ^36^ ^]^ Among them, the upregulation or amplification of AKT2 is frequently observed in OC cells, which is associated with more severe cancer cell invasion, metastasis, and chemoresistance.^[^ ^37^, ^38^, ^39^ ^]^ In contrast, AKT1 expression levels are more frequently elevated in gastric carcinoma,^[^ ^40^ ^]^ and *AKT3* mRNA expression is upregulated in estrogen receptor‐negative breast carcinomas.^[^ ^41^ ^]^ In addition to the varying distribution of AKT isoforms, previous studies have demonstrated opposite roles of AKT1 and AKT2 in tumorigenesis. While AKT1 inhibits cell migration and invasion, AKT2 has an opposite effect.^[^ ^42^, ^43^ ^]^ These observations, together with ours, strongly suggest that AKT2 is the main pro‐oncogenic kinase in OC. Moreover, it was suggested that the multimeric complexes formed by AKT proteins are confined to individual isoforms^[^ ^44^ ^]^ In our study, we showed that PAD1 specifically interacts with AKT2 and not with any other AKT isoforms, consistent with a previous study in gliomas showing that astrocyte elevated gene‐1 (AEG‐1) protein also specifically interacts with AKT2 and not with AKT1 or AKT3.^[^ ^45^ ^]^ Moreover, the potential role of PAD1 in maintaining the stemness of OCSLCs was partially clarified when our finding that citrullination of AKT2 by PAD1 promotes AKT2 kinase activity in OCSLCs is put in the context of the above roles of AKT2 in tumorigenesis. Consistent with these findings, an analysis in breast cancer demonstrated that AKT2 overexpression was correlated with the acquisition of cancer stem‐like properties, responsible for invasiveness, chemoresistance, and poor cancer outcomes.^[^ ^46^ ^]^ Overall, our findings suggest that the citrullination of AKT2 catalyzed by PAD1 may be critical in coordinating events related to the maintenance of OCSLC stemness and the acquisition of drug resistance, thereby constituting the PAD1/AKT2 pathway as a potential therapeutic target for novel interventions in OC.

It is widely acknowledged that citrullination, an important posttranslational modification of arginine residues, influences a broad spectrum of cellular biological functions, including those of cancer cells.^[^ ^33^, ^47^ ^]^ Due to the loss of charge in substrate proteins, citrullination can either regulate the interactions with other proteins and nucleic acids or alter the activity of the target proteins. For example, citrullination of histone tails catalyzed by PAD2 and PAD4 can induce local alterations in the chromatin structure and modulate tumor‐associated gene transcription in human cancer cells.^[^ ^34^, ^48^, ^49^ ^]^ PAD2‐mediated citrullination of a non‐histone protein, RNA polymerase II (RNAP2), can facilitate the interaction between RNAP2 and the p‐TEFb complex, leading to gene transactivation and breast cancer cell proliferation.^[^ ^50^ ^]^ In our previous study, we demonstrated that PAD2‐catalyzed citrullination of MEK1 enhanced MEK1 kinase activity and promoted downstream ERK1/2 activation in endometrial cancer cells, although this modification did not seem to affect its own phosphorylation.^[^ ^24^ ^]^ In line with the citrullination activity detailed in these studies, we identified AKT2 as a novel target of PAD1 in OCSLCs and found that PAD1 specifically catalyzes the citrullination of AKT2 at the R202 residue within the AKT2 kinase domain. Either depletion of PAD1 or inhibiting PAD1 enzymatic activity reduced the phosphorylation level of AKT2. Importantly, mutation of AKT2 R202 also resulted in a comparable inhibitory effect, suggesting that phosphorylation of AKT2 relies on the enzymatic activity of PAD1. Further, PAD1‐catalyzed citrullination of AKT2 is coupled with the kinase activity of AKT2 to activate CEBPβ expression, leading to an increase in the expression of OC stemness‐related markers, and facilitating the maintenance of OCSLC stemness.

To the best of our knowledge, this is the first report demonstrating that citrullination of a kinase can influence its phosphorylation and subsequent kinase activity. In contrast to our previous findings in endometrial cancer, which showed that citrullination of MEK1 had no effect on its own phosphorylation,^[^ ^24^ ^]^ citrullination of AKT2 was essential for its phosphorylation at S474 and T309, two critical residues required for AKT2 activation. Given that citrullination can neutralize positively charged arginine residues in the substrate and alter protein structure, and that our protein structure models indicated that only the R202 mutation disrupted the exposure of S474 or T309 in AKT2, we believe that PAD1‐mediated AKT2 citrullination at R202 might lead to a more favorable exposure of T309 and S474 for phosphorylation in AKT2. It is also possible that citrullination of AKT2 helps maintain the proper AKT2 conformation, allowing upstream phosphatidylinositol‐dependent kinase 1 (PDK1) and mTOR complex 2 (mTORC2) to have closer access to AKT2. Hyperactivation of AKT is closely associated with unfavorable prognoses and chemotherapeutic resistance in human cancers.^[^ ^51^, ^52^ ^]^ A previous study demonstrated that phosphorylation of S477 and T479 at the carboxy terminus of AKT1, which promotes S473 phosphorylation, offers an essential interpretation for the full activation of AKT1.^[^ ^53^ ^]^ Therefore, we cannot exclude the possibility that citrullination of AKT2 at R202 might also play a role in AKT2 hyperactivation. Besides, it has been reported that phosphorylation of S473 in AKT1 could assist in protecting AKT1 from dephosphorylation,^[^ ^54^ ^]^ whereas the interaction between AKT2 and other binding proteins could prolong the stability of AKT2 phosphorylation at S474.^[^ ^45^ ^]^ Given that citrullination of AKT2 promotes its phosphorylation at S474, it is possible that citrullination‐enhanced phosphorylation of AKT2 contributes to stabilizing the active conformation of AKT2. These findings warrant further investigation.

Typically, a combination of multiple inhibitors targeting the same molecular target can elicit a pronounced synergistic effect compared to a single inhibitor treatment.^[^ ^55^ ^]^ Our results just offer such a solution that inhibiting PAD1‐catalyzed AKT2 citrullination compromises AKT2 activation in OCSLCs, which may provide a novel target for regulating the activation of the AKT pathway. This also holds potential for a therapeutic approach involving the combination of PAD1 and AKT2 inhibitors for targeting OCSLCs. Together, our findings not only lay a theoretical foundation for the combined application of PAD1 and AKT inhibitors, but also provide novel research perspectives on how to effectively reverse chemotherapy resistance in the treatment of OC.

## 4 Experimental Section

### Cell Culture and Treatment

OVCAR3 and HEK 293 cells were cultured in DMEM supplemented with 10% fetal bovine serum (Gibco, USA) at 37 °C under 5% CO_2_. SKOV3 cells were maintained in RPMI‐1640 medium supplemented with 10% fetal bovine serum at 37 °C in a humidified 5% CO_2_ incubator. PAD1‐depleted OVCAR3 and SKOV3 cells were generated by transduction with mission lentiviral transduction particles containing a short hairpin RNA (shRNA) construct targeting the human PAD1 coding sequence (Sigma SHCLND_NM_01 3358). In the control group, cells were transduced with a non‐targeting shRNA lentiviral construct (Sigma SHC002V). Cells were selected in medium containing 1 µg mL^−1^ puromycin (Sigma, USA). For CRISPR/Cas9‐mediated PAD1 or AKT2 knockout (KO) in OVCAR3 cells, two sgRNAs (sequences summarized in Table S5, Supporting Information) were cloned into the pGL3‐U6‐sgRNA vector. Cells were co‐transfected with CRISPR sgRNA plasmids and the pST1374‐N‐NLS‐flag‐linker‐Cas9 plasmid. Both vectors were kindly provided by Dr. Bin Sheng (Nanjing Medical University, Nanjing, China). Stable PAD1‐KO or AKT2‐KO cells were selected using medium supplemented with 1 µg mL^−1^ puromycin and 2 µg mL^−1^ blasticidin (Invitrogen). For siRNA transfection, three independent siRNAs were transfected into OVCAR3 cells using Lipo3000 (Lipomaster 3000 Transfection Reagent, TL301, Vazyme, China) at a final concentration of 50 nM. After 8 h, the medium was replaced with fresh medium, and the cells were harvested 48 h post‐transfection for subsequent analysis. The cisplatin‐resistant OVCAR3‐CisR cells were successfully selected through exposing the parental OVCAR3 cells to an increasing concentration of 2 µM cisplatin for 2 months, followed by 5 µM cisplatin treatment for another 3 months. Where indicated, D‐Cla was diluted in cell culture medium at the final concentration of 0, 50, 100, and 200 µM, and added to cells for 48 h before harvest. LY294002 was diluted in cell culture medium at final concentrations of 0, 5, 10, and 20 µM, and added to cells for the indicated times before harvesting. AZD5363 was diluted in cell culture medium at a final concentration of 5 µM, and added to cells for 48 h before harvesting.

### Cell Proliferation Assay

Cell proliferation assays were performed using a Cell Counting Kit‐8 (CCK‐8) from Vazyme, China (A311‐02). Briefly, 5000 cells well^−1^ were plated in triplicate in a 96‐well plate. When indicated, cells were treated with either D‐Cla or LY294002. Following incubation with the dye solution at 37 °C in the dark for 2 h, the formazan product was measured at 450 nm. The absorbance values in different groups can indirectly reflect cell viability.

### Transwell Invasion Assay

Transwell invasion assays were performed in 24‐well plates with 8‐µm pore size chamber inserts (Corning, USA), according to the protocols recommended by the manufacturer. Briefly, the upper surface of the filter was coated with 50 µL Matrigel (Corning, 356 243) diluted 1:3 in serum‐free DMEM. ≈50 000 single cells in serum‐free DMEM were added to the upper chamber of the filter membrane. The lower compartment of the Transwell chamber was filled with 500 µL complete media. After 48 h, the cells on the lower surface were fixed with methanol and stained with 0.1% crystal violet. Representative photographs were taken in three independent fields for each well under a light microscope. Image J software (National Institute of Mental Health, MA, USA) was used to count the cells that penetrated the Matrigel. The number of cells penetrating the Matrigel reflects the relative invasiveness of OC cells.

### Wound‐Healing Assay

OVCAR3 and SKOV3 cells were seeded in 6‐well plates and grown to full confluence in complete media, with three parallel wells for each condition. The monolayer of cells was scratched with a 10 µL pipette tip, and washed twice with serum‐free DMEM to remove the detached cells. The wounded areas were observed and imaged under a microscope. Cell distances were imaged 48 h after scratching. The changes in cell migration were determined by comparing the difference in wound‐healing areas at least at four fields using ImageJ. The data from three independent experiments were used to calculate of the final values.

### Fluorescence‐Activated Cell Sorting

About 50 000 OVCAR3 cells were resuspend in 500 µL antibody incubation solution, and 2 µL PE‐CD133 antibody was added. After incubation in the dark for 30 min, the cells were sorted under sterile conditions. The obtained CD133‐positive (CD133^+^) or CD133‐negative (CD133^−^) cells were continuously cultured.

### Mammosphere Formation Assay

≈1000 OVCAR3 cells, as single cell suspensions per well were seeded in an ultralow attachment flat bottom 6‐well plate (Corning 3471) using tumor stem cell culture medium (1 × B27 + 20 ng mL^−1^ bFGF + 20 ng mL^−1^ EGF). After 2 weeks of cell culture, the tumor spheroids formed were observed under a light microscope, and only those mammospheres larger than 100 µm in diameter were counted.

### Immunohistochemistry Analysis

Tumor samples were collected from the Department of Pathology, the First Affiliated Hospital of China Medical University, China. All samples were collected according to patients’ informed consent and the study was carried out according to the Institute ethics guidelines (Approval number: CMU[2024]192). Tissue sections were deparaffinized, rehydrated, and then incubated for 20 min in 3% hydrogen peroxide to quench the endogenous peroxidase activity. Sections were then heated to retrieve the antigen in 0.01 m citrate buffer (pH 6.0) for 15 min and then blocked with 10% goat serum in PBS. The immunohistochemical analysis was performed using a immunohistochemistry staining kit (PV‐9000, ZSGB‐BIO, China) with primary antibodies at 4 °C overnight. Sections stained were examined using a Zeiss Axio Observer microscope. Nonimmunized rabbit or mouse IgG served as negative control. The antibodies used are listed in Table S1, Supporting Information.

### Immunofluorescence Staining

OC cells were seeded on glass slides. After washing with PBS, cells were fixed with 4% paraformaldehyde for 20 min, permeabilized with 0.1% Triton X‐100 for 10 min, and blocked with 5% BSA in PBS for 2 h at room temperature. Primary antibodies were added to the cells overnight at 4 °C. Fluor 488‐conjugated goat anti‐mouse or Fluor 546‐conjugated secondary antibodies were used to visualize the location and expression intensity of the target protein. DAPI (Vector Laboratories, Cambridgeshire, UK) was used to locate the location of the cell nucleus. Representative images were collected with LSM 700 laser scanning confocal microscope (Carl Zeiss). The antibodies used are in Table S1, Supporting Information.

### Western Blot

The cells were washed twice with cold PBS and lysed in cold radioimmunoprecipitation assay (RIPA) buffer (150 mM NaCl, 50 mM Tris‐HCl, pH 7.4, 1% sodium deoxycholate, 1% Triton X‐100, and 0.1% SDS) containing protease inhibitors for 30 min. The lysates were then centrifuged and the supernatants were collected. The total proteins were denatured and separated by SDS‐PAGE gel, and then transferred to the PVDF membrane. The membranes were blocked with 5% non‐fat milk in Tris‐buffered saline containing 0.1% Tween‐20 (TBST) for 2 h at room temperature. The membranes were then incubated with primary antibodies overnight at 4 °C. GAPDH was used as a loading control. The membranes were washed and then incubated with horseradish peroxidase‐conjugated secondary antibodies. The signal was visualized using an Enhanced Chemiluminescence Detection Kit (Pierce Biotechnology, USA). The antibodies used are listed in Table S1, Supporting Information.

### Antibody Preparation

A site‐specific citrulline antibody against AKT2 R202 citrulline was generated at AtaGenix Laboratory (Wuhan, China). Briefly, two rabbits were immunized with AKT2 peptides (aa 193–207) citrullinated at residue 202 (Cys + EVAHTVTES_Cit_VLQNT) which were C‐terminally coupled to KLH. 2 weeks post the initial immunization, three booster injections were administered every 2 weeks. Crude sera of the final bleed were collected for antibody purification. Besides, 5 mg of citrullinated (same as immunogen) and unmodified (Cys+EVAHTVTES_R_VLQNT) AKT2 peptides were used to prepare antigen affinity chromatography. The crude sera from the two rabbits were purified separately. The antisera were purified by citrullinated peptide affinity column, and the cross reaction was removed by unmodified peptide affinity column. The purified antibodies were eluted in elution buffer (0.1 M glycine HCl pH 3.0), and ice‐cold neutralization buffer (1 M Tris HCl pH 8.5) was added and mixed thoroughly to neutralize the pH to 7.2.

### Dot Blot

Citrullinated and unmodified AKT2 peptides were coated on nitrocellulose membrane and incubated at 37 °C for 2 h. After blocking in 5% non‐fat milk at room temperature for 2 h, the membrane was incubated with the anti‐AKT2 Cit202 antibody for 1 h at room temperature. The membranes were washed five times with TBST and then incubated with horseradish peroxidase‐conjugated rabbit IgG for 1 h at room temperature. The signals were visualized using an Enhanced Chemiluminescence Detection Kit (Pierce Biotechnology, USA).

### Immunoprecipitation Assay

HEK 293 cells were transfected with Flag‐tagged PAD1 in pcDNA3.1 (+) together with HA‐tagged AKT1, AKT2, or AKT3 using Lipomaster 3000 transfection reagents (TL301‐02, Vazyme, China). Whole cell lysates were immunoprecipitated with anti‐Flag antibody or anti‐HA antibody. Immunoprecipitates were washed and analyzed by western blot using anti‐HA and anti‐Flag antibodies as indicated. To test the endogenous binding of PAD1 to AKT2, the whole cell lysates of OVCAR3 cells were immunoprecipitated with anti‐PAD1 antibody, and the immunoprecipitates were washed and detected by western blot using anti‐AKT1, anti‐AKT2, and anti‐AKT3 antibodies (Table S1, Supporting Information).

### PAD Enzymatic Activity Assay

AKT2 proteins were overexpressed and purified from HA‐tagged AKT2 in pcDNA3.1(+) using an anti‐HA antibody combined with rProtein A/G Agarose Resin (#36 403, Yeasen). PAD1 proteins were overexpressed and purified from Flag‐tagged PAD2 in pCMV using anti‐Flag M2 affinity gel system (Sigma). The purified AKT2 was treated with PAD1 in PAD buffer containing 50 mM Tris‐HCl, pH 7.6, 4 mM DTT, 4 mM CaCl_2_ at 37 °C for 6 h. Citrullination of AKT2 was detected either using an anti‐Citrulline (Modified) Detection Kit (#17‐347, Millipore, USA), or an anti‐AKT2 Cit202 antibody.

### Luciferase Reporter Assay

The proximal promoter regions of *SOX2, CD44*, and *CD133* were cloned and introduced into a modified pGL3 luciferase reporter vector. The firefly luciferase vector was used for internal control. The constructs were confirmed by sequencing. Then a mixture of luciferase reporter plasmid and Flag‐CEBPβ plasmid or the corresponding controls were co‐transfected into HEK293 cells. Cells were harvested after 48 h of transfection. The luciferase activity was measured by the Luciferase Reporter Assay System (#E2920, Promega, USA), normalized to an equal quantity of protein, as measured by the Bradford assay.

### Protein Interaction Simulation

3D crystal structures of PAD1 and AKT2 protein were obtained through homology modeling. These crystal structures were then subjected to protein preprocessing, regenerating states of the native ligand, H‐bond assignment optimization, protein energy minimization, and water removal using the Protein Preparation Wizard module in Schrödinger Maestro 13.5. Protein‐protein interaction simulation was performed with molecular docking module (Number of ligand rotations to probe = 70 000 and Maximum poses to return = 30). The lower the protein‐protein interaction score, the lower the binding free energy of the two proteins, and the higher the binding stability. The Protein Interaction Analysis module was used to determine the specific regions where PAD1 binds to AKT2 protein.

### RNA Extraction and Quantitative Real‐Time PCR Assay

Total RNA was extracted using the total RNA Extraction Reagent (R401‐01, Vazyme), followed by DNase treatment. The first strand of cDNA was synthesized by the HiScript first Strand cDNA Synthesis Kit (R111‐01, Vazyme). Quantitative real‐time PCR was performed using the Power SYBR Green PCR Master Mix (Q711‐02, Vazyme) with gene‐specific primers. The primers used were listed in Table S2, Supporting Information. All target gene transcripts were normalized to *GAPDH*, with the exception of *HIF‐1α*, which was normalized to *β‐ACTIN*. The relative fold change in expression was calculated using the 2^−ΔΔCT^ method.

### RNA‐Seq Analysis

Total RNA was extracted from *PAD1*‐knockdown, AKT2 WT or R202K overexpressed, and the corresponding control OVCAR3 cells using RNA Extraction Reagent. Libraries preparation and sequencing were performed by Annoroad Gene Technology (Beijing China). The index codes were added to attribute sequences to each sample. Each group was sequenced in duplicate. The library preparations were sequenced on an Illumina Hiseq platform and 150 bp paired‐end reads were generated. The quality‐controlled clean reads were aligned to the reference human genome hg38 using HISAT2. Featurecount was used to count the reads mapped to each gene. Differential gene expression analysis between two comparison groups (two biological replicates per group) was performed using DESeq2. Genes with *p*‐value < 0.05, log_2_(FoldChange) > 1 and Count > 10 were considered differentially expressed. The GO enrichment analysis of differentially expressed genes is realized by clusterProfiler, the biological process is selected and the GOBubble graph is drawn by R packages named the ggplot2 and GO plot data. GSVA analysis uses the gene set in the MSigDB database to evaluate whether different metabolic pathways are enriched among different samples. The results are visualized in a heatmap, generated by the pheatmap package. Raw FASTQ files for the RNA‐seq libraries are deposited in the NCBI Sequence Read Archive (SRA) and have been assigned BioProject accession: PRJNA1173848.

### Chromatin Immunoprecipitation and ChIP‐qPCR

Before harvest, cells were crosslinked with a final concentration of 1% paraformaldehyde for 10 min at 37 °C. Crosslinking was quenched in 125 mM glycine on ice for 5 min. The cell lysates were sonicated and centrifuged. The supernatant was collected and diluted tenfold in dilution buffer, and pre‐cleared with rProtein A/G Agarose Resin (#36 403, Yeasen). The precleared, chromatin‐containing supernatant was used in immunoprecipitation reactions with antibodies against CEBPβ, using non‐specific rabbit IgG as a control. A fraction 10% of the supernatant was saved as reference control. The immunoprecipitated genomic DNA was cleared of protein and residual RNA by digestion with proteinase K and RNase (Roche), respectively. The DNA was then extracted with phenol:chloroform:isoamyl alcohol and precipitated with ethanol. For gene‐specific ChIP analysis, quantitative real‐time PCR (qPCR) was used to determine the enrichment of immunoprecipitated DNA relative to the input DNA using gene‐specific primer sets (Table S3, Supporting Information). Each ChIP experiment was conducted a minimum of three times with independent chromatin isolates to ensure reproducibility. The antibodies were listed in the Table S1, Supporting Information.

### Mass Spectrometry and Identification of Citrullinated Peptides

The MS analysis was performed at Aimsmass Co. (Shanghai, China). Briefly, the PAD1‐treated AKT2 bands were excised from the gel and treated with 10 mM DTT for 45 min at 57 °C, followed by re‐acting with 15 mm iodoacetamide for 45 min in the dark. The sample was then digested with trypsin. The peptide samples were then desalted and lyophilized. The samples were then resuspended with 0.1% formic acid and loaded onto an HPLC chromatography system, Fisher Easy‐nLC 1000 equipped with a C18 column. Peptides were separated by an HPLC gradient (4–18% buffer B in 182 min; and 18–90% in 13 min at a flow rate of 300nL min−1; buffer A = 0.1% formic acid, buffer B = 100% acetonitrile). Mass spectrometry analysis was carried out in the positive‐ion mode with full scans (350–1600 m z^−1^) at a mass resolution of 30 000. The MS/MS spectra were searched using the software Proteome Discoverer 1.4 (Thermo). The fragment mass tolerance was set to 0.05 Da. Carboxyamidomethylation of cysteine (+57.021 Da) was set as a fixed modification, whereas citrullination of Arg (+0.9848 Da) was set as a variable modification. All MS/MS analyses indicating citrullinated peptide fragments were then manually confirmed.

### Mutagenesis

Point mutations in the constructs were mutated using QuikChangemulti Site‐Directed Mutagenesis kit (200 514, Agilent), or MutExpress II Fast Mutagenesis Kit V2 (C214, Vazyme) according to the manufacturer's instructions. Mutations were confirmed by Sanger sequencing. Mutation primers are provided in Table S4, Supporting Information.

### Xenograft Tumor Model in Nude Mice

All animal experiments were approved by the Institutional Animal Care and Use Committee of the China Medical University (Approval number: CMU20242016). 6‐week‐old female BALB/c nude mice were purchased from Beijing Vital River Laboratory Animal Technology, and maintained in a special pathogen‐free environment. Where indicated, 1 × 10^7^ PAD1‐depleted OVCAR3 cells were injected subcutaneously on the left flanks, and the corresponding control cells on the right flanks of nude mice. ≈18 days post‐inoculation, a visible and palpably firm solid tumor forms subcutaneously in nude mice. To evaluate the effect of the combination of Cisplatin and D‐Cla on tumor growth, OVCAR3 cells were inoculated subcutaneously into the axilla of 5 nude mice on both sides and CisR OVCAR3 cells were inoculated into 10 nude mice in the same way. The 10 mice were randomly divided into 2 groups. When indicated, 5 mg kg^−1^ of Cisplatin, or 5 mg kg^−1^ of Cisplatin combined with 20 mg kg^−1^ of D‐Cla were intraperitoneally injected into the nude mice once every 3 days. Tumor diameters are measured with digital calipers, and the tumor volume in mm^3^ was calculated by the following formula: Volume = 0.5 × (width)^2^ × length. Mice were sacrificed under anesthesia 6 weeks post cell inoculation or 4 weeks post drug injection, and the tumors were harvested. For extreme limiting dilution assay (ELDA), four dilutions of OVCAR3‐derived cells (1 × 10^7^, 1 × 10^6^, 1 × 10^5^, and 1 × 10^4^ cells) were injected into nude mice (n = 6/group). 4 weeks post inoculation, mice were euthanized for collection of tumor tissues. ELDA software (http://bioinf.wehi.edu.au/software/elda/index.html) was applied to calculate cancer stem‐like cell frequency. For intraperitoneal metastasis experiment, PAD1‐depleted or AKT2 R202K mutant OVCAR3 cells were injected intraperitoneally (5 × 10^6^ cells per mouse), and the tumor burden was assessed after 66 days (three mice per group). At the experimental endpoint, ascites was extracted from the peritoneal cavity using a syringe, and the volume was measured. Additionally, the number of tumor nodules formed in the peritoneal cavity was recorded.

### Statistical Analysis

All experiments were carried out at least three biological replicates, and the experimental data obtained were expressed as mean ± standard deviation. Statistical evaluation for data analysis was determined by Student's *t*‐test or one‐way ANOVA with *p < 0.05, **p < 0.01 indicating significantly different from control.

## Conflict of Interest

The authors declare no conflict of interest.

## Author Contributions

T.X. and X.Q.L. contributed equally to this work. X.Z. and X.Q.L. designed the study and wrote the paper; T.X. contributed to the experiments, interpretation, and data analysis; C.S., S.F., and J.G. helped with experimental operation, and data acquisition; F.L., Y.H., and J.X. analyzed the clinical data; P.T. contributed to the PAD1 inhibitor.

## Supporting information



Supporting Information

## Data Availability

The data that support the findings of this study are available in the supplementary material of this article.
