# Supplementary material for: Citrullination of AKT2 Catalyzed by PAD1 Facilitates the Maintenance of Stemness Characteristics of Ovarian Cancer Stem‐Like Cells in Ovarian Cancer
Source: Adv Sci (Weinh). 2025 Aug 22;12(39):e01014. doi: 10.1002/advs.202501014 (PMC12533369; doi:10.1002/advs.202501014)
Supplement: Supplementary file 1 — Supporting Information [file ADVS-12-e01014-s001.doc]

**SUPPLEMENTAL INFORMATION**

**Citrullination of AKT2 Catalyzed by PAD1 Facilitates the Maintenance of Stemness Characteristics of Ovarian Cancer Stem-Like Cells in Ovarian Cancer**

Teng Xue, Xiaoqiu Liu, Chao Song, Shujia Fei, Jian Gu, Yun Han, Jia Xing, Xiaohan Liu, Fei Liang, Paul R Thompson, Xuesen Zhang

Figures S1-S9

Supplementary Tables 1-6

**Figure S1**


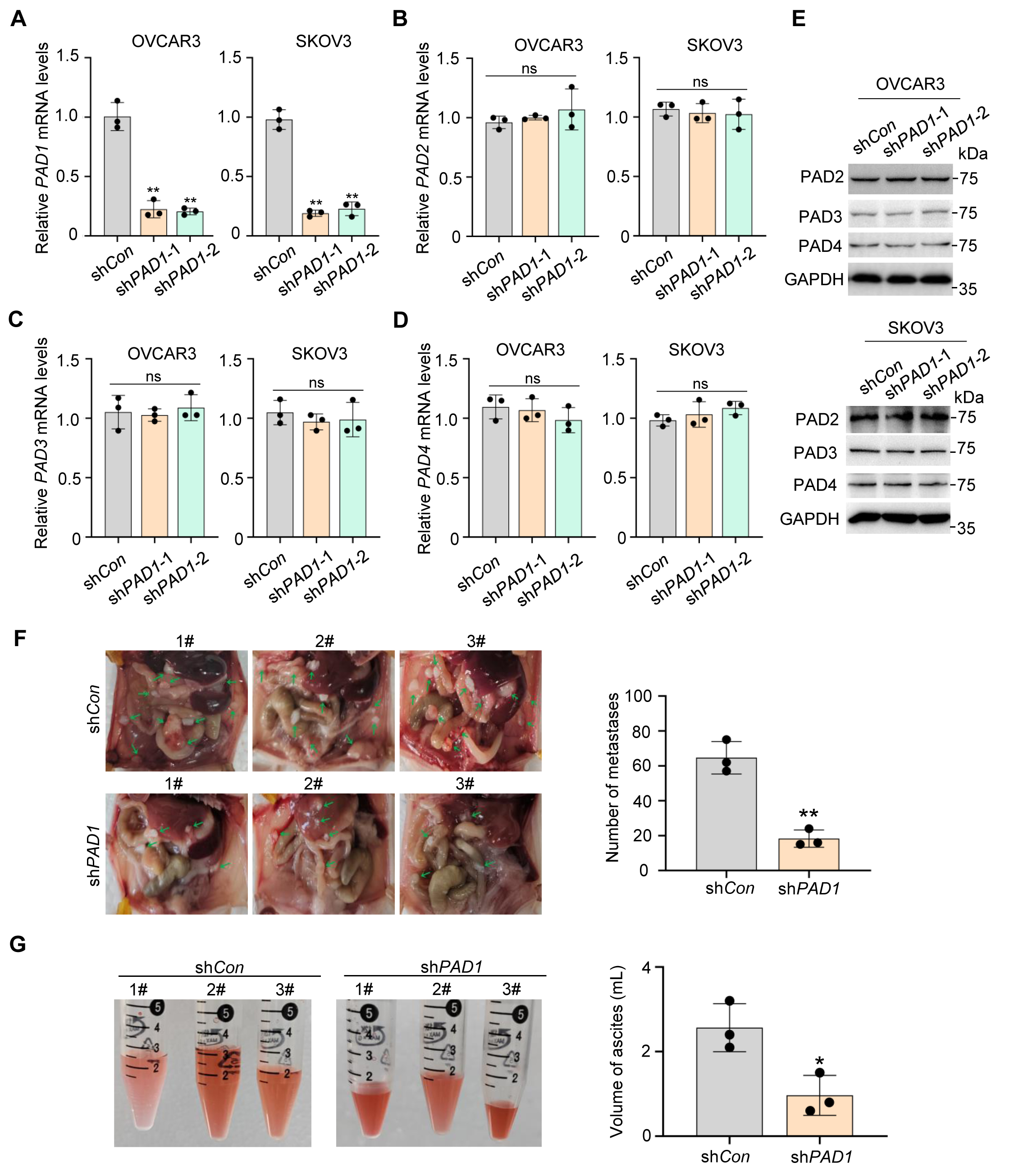


**Figure S1 Related to Figure 1**

**A-D)** qRT-PCR analysis of *PAD1* (A), *PAD2* (B), *PAD3* (C), *PAD4* (D) mRNA levels upon PAD1 knockdown in both OVCAR3 and SKOV3 cells. sh*PAD1*-1 and sh*PAD1*-2 represent two individual *PAD1* shRNA vectors. *GAPDH* was used as the reference control.

**E)** Western blot analysis of PAD2, PAD3, PAD4 protein expression levels in OVCAR3 and SKOV3 cells with stable PAD1 knockdown. GAPDH served as the loading control.

**F)** Intraperitoneal xenograft tumor formation in nude mice derived from PAD1 knockdown OVCAR3 cells and control cells. The arrow indicates macroscopically visible liver, peritoneal and mesenteric metastasis (Left). Statistical analysis was performed on the number of metastasis (Right).

**G)** The volume of ascites (Left) and statistical analysis (Right) from the mice treated as described in Figure F.

Results are presented as mean ± SD, n = 3. **p* <0.05, ***p* < 0.01. A-D: one-way ANOVA; F, G: Student’s t-test.

**Figure S2**


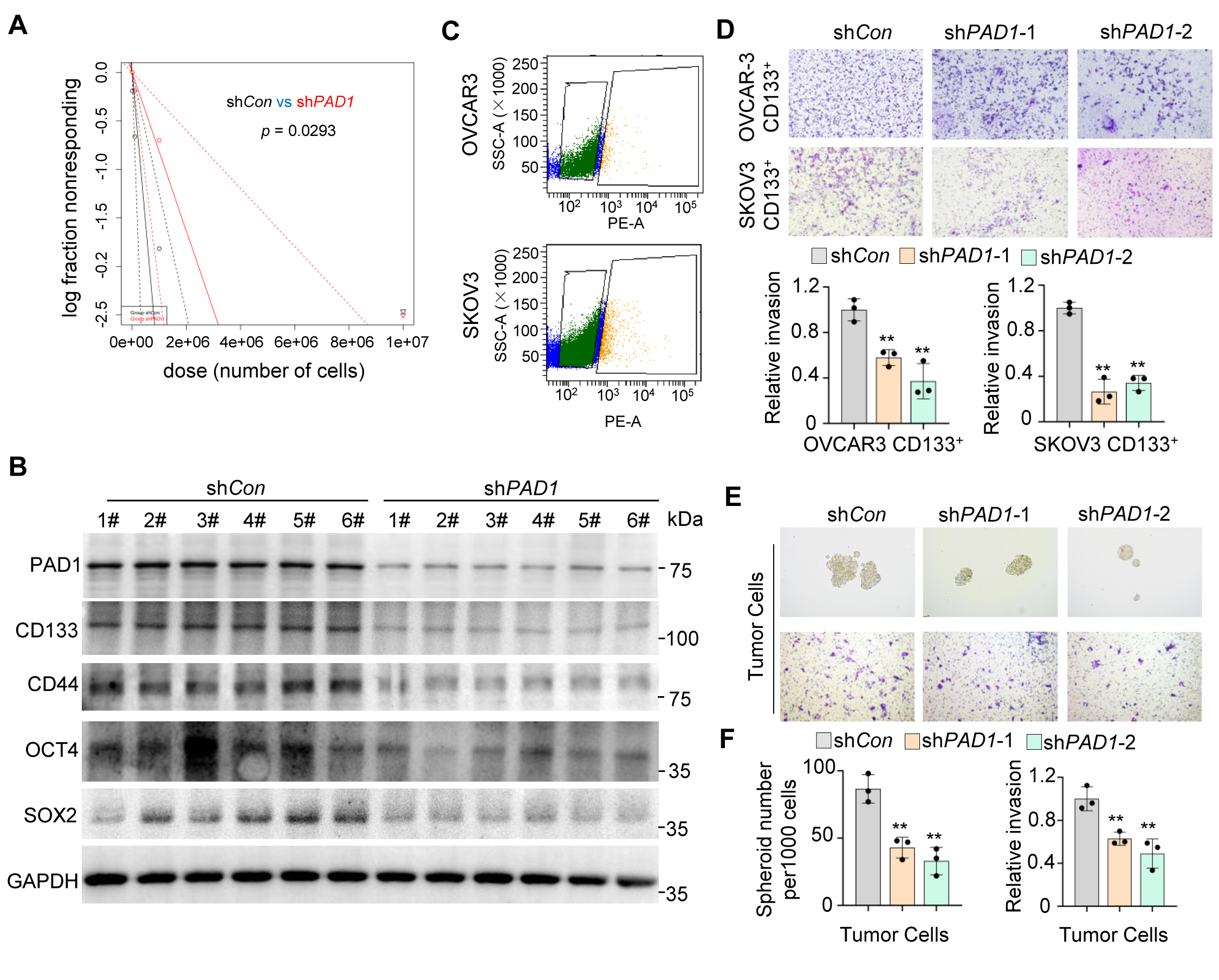


**Figure S2**  **Related to Figure 2**

**A)** The OCSLC frequency analysis was calculated by extreme limiting dilution analysis (ELDA) software (<http://bioinf.wehi.edu.au/software/elda>). A plot of the log fraction of nonresponding versus the number of cells, the slope of the line representing the estimated log‐OCSLC fraction and the dotted lines give the 95% confidence interval.

**B)** Protein levels of PAD1, CD133, CD44, OCT4, and SOX2 in tumor lysates. GAPDH served as a loading control.

**C)** FACS plot depicting the gating strategy used to sort CD133^+^ cells from both OVCAR3 (top) and SKOV3 cells (bottom). SSC: Side Scatter; PE: phycoerythrin.

**D)** Representative images (top) and quantification (bottom) for transwell assay in PAD1 KD OVCAR3 (CD133^+^ subpopulation) and PAD1 KD SKOV3 cells (CD133^+^ subpopulation), compared to the respective shRNA control cells.

**E, F)** Representative images (E) and quantification (F) for tumor cell-derived spheroid from ascites of ovarian cancer patients with diameters greater than 100 μm cultured in stem cell culture medium in flat bottom ultra-low attachment plates without FBS (top) and transwell assay (bottom) upon PAD1 knockdown. Results are presented as mean ± SD, n = 3. ***p* < 0.01. D, F: one-way ANOVA.

**Figure S3**

**
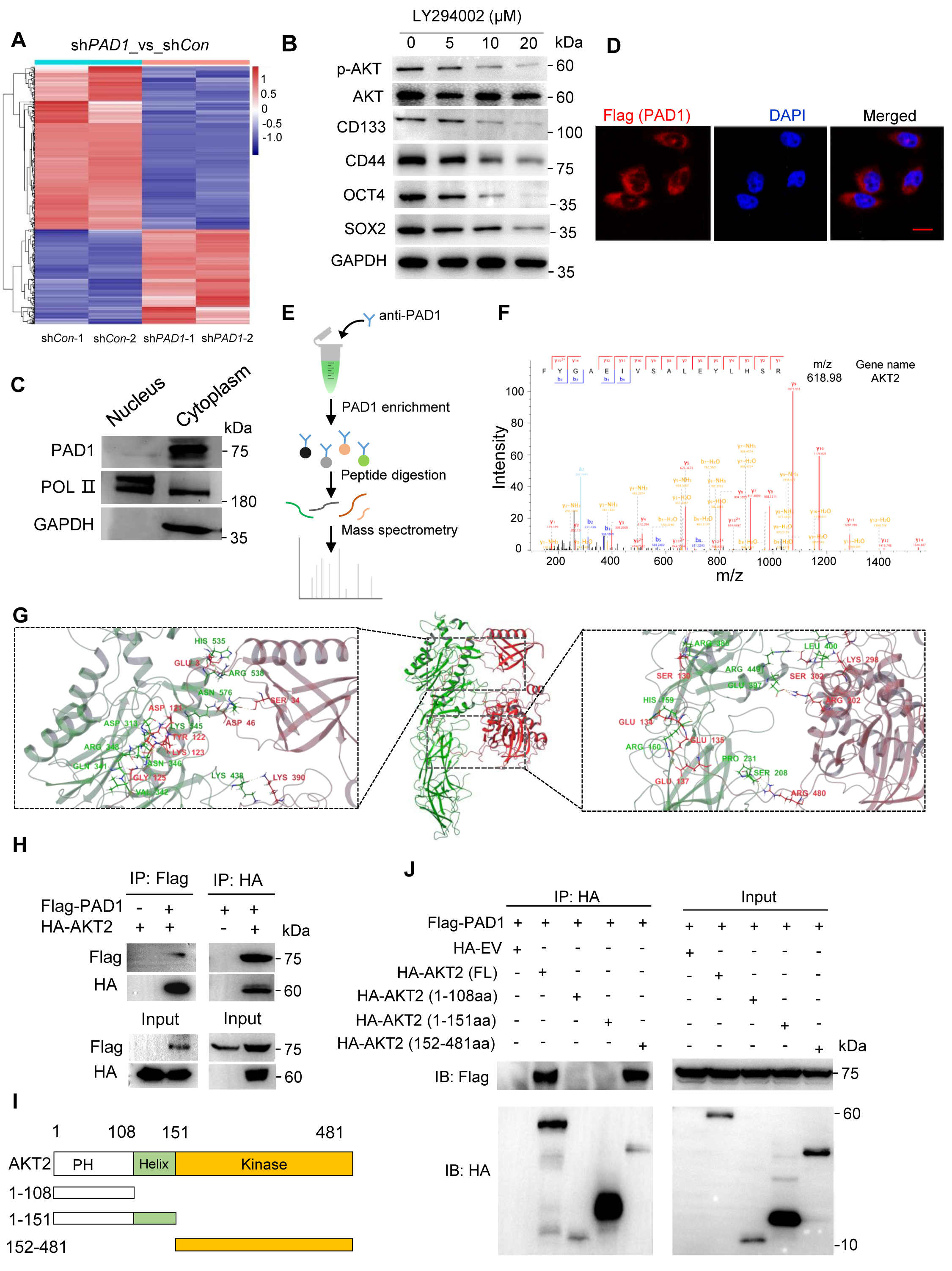
**

**Figure S3 Related to Figure 3**

**A)** Heatmap showing the relative expression levels of genes differently expressed between PAD1 knockdown and control OVCAR3 cells.

**B)** Western blot analysis of p-AKT, AKT, CD133, CD44, OCT4, and SOX2 in OVCAR3 cells treated with LY294002 at the indicated dose. GAPDH served as the loading control.

**C)** Cellular proteins from OVCAR3 cells were separated into cytoplasmic and nuclear pools by fractionation methods and examined by Western blot with anti-PAD1 antibody. Cleanliness of fractionation was determined by probing with antibodies for Pol II (nuclear) and GAPDH (cytoplasmic) proteins.

**D)** Representative immunofluorescence images showing the overexpressed PAD1 (Flag-tagged) in HEK293 cells. Cells were stained with antibody against the Flag-tag (red). Nuclei were stained with DAPI. Bar=5 µm.

**E)** Schematic diagram of identification process of PAD1 binding protein in OVCAR3 cells.

**F)** MS/MS analysis identifies AKT2 protein peptide from the PAD1 immunoprecipitates in OVCAR3 cells.

**G)** Molecular docking model for the interaction of PAD1 (green) with AKT2 (red). A close-up view showing the interfacial regions between PAD1 and AKT2. The GLU3 residue of AKT2 establishes a hydrogen bond with the ARG538 residue of PAD1, while the ASP46 residue of AKT2 forms both a hydrogen bond and a salt bridge with LYS545 from PAD1. Additionally, the ARG202 residue of AKT2 engages in two hydrogen bonds and one salt bridge with GLU397 from PAD1.

**H)** Reciprocal co-immunoprecipitation analysis of the interaction between PAD1 and AKT2 proteins in HEK293 cells overexpressed Flag-tagged PAD1 and HA-tagged AKT2.

**I)** Schematic diagram of truncated forms of ATK2.

**J)** Co-immunoprecipitation analysis of the interactions between Flag-tagged PAD1 and HA-tagged AKT2 constructs, including full-length HA-tagged AKT2 (FL), HA-tagged AKT2 (1-108 aa), HA-tagged AKT2 (1-151 aa), and HA-tagged AKT2 (152-481 aa), in HEK293 cells.

**Figure S4**


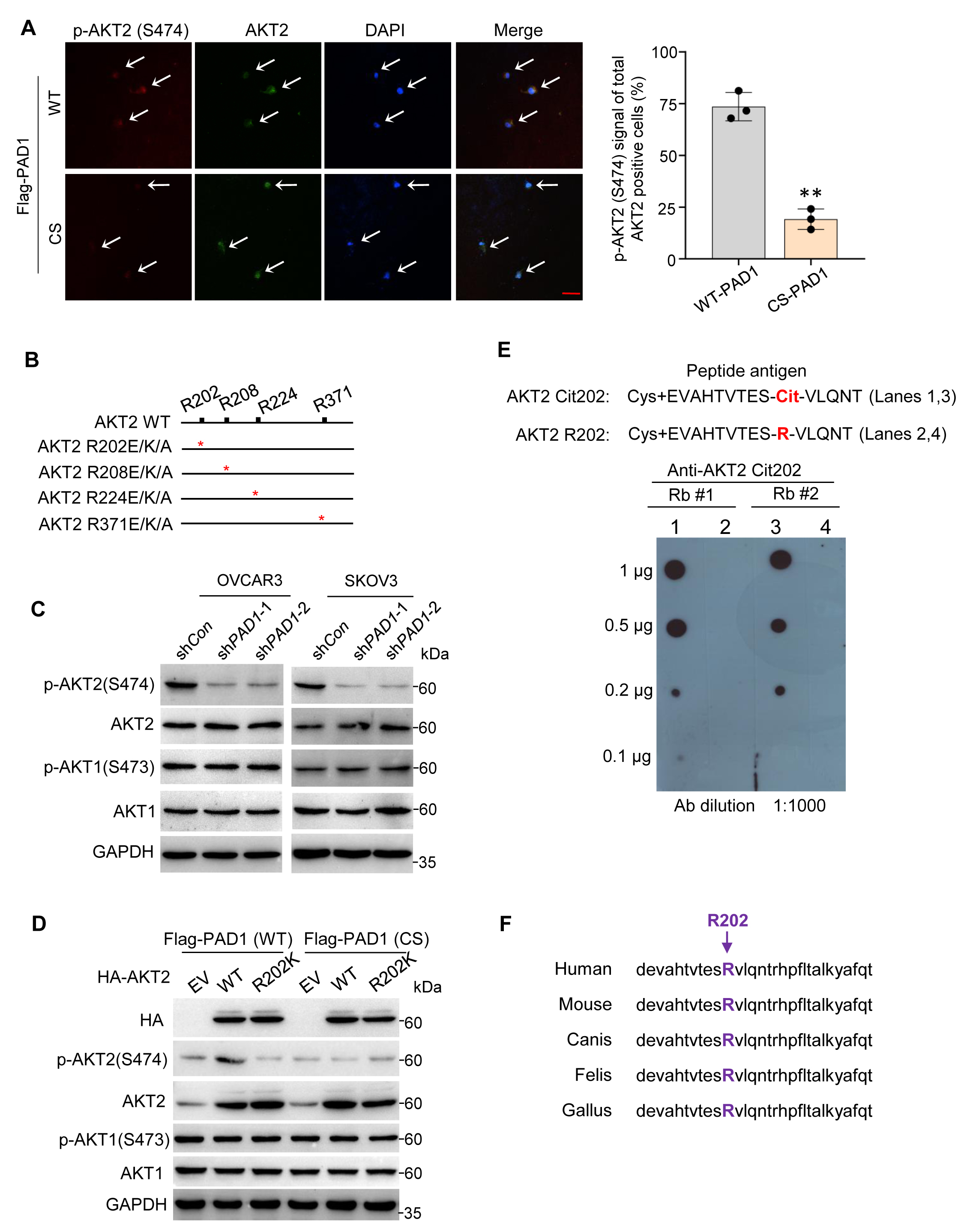


**Figure S4 Related to Figure 4 and 5**

**A)** Representative immunofluorescence images (Left) and quantification (Right) of co-localization of p-AKT2 (S474) and AKT2 in OVCAR3 cells overexpressing Flag-PAD1 (WT or CS). Arrows indicating PAD1-positive cells. Bar =20 μm.

**B)** Schematic illustration of RK mutation of AKT2. Red stars indicating the corresponding mutant arginine sites.

**C)** Western blot analysis of p-AKT2 (S474) and p-AKT1 (S473) in PAD1 KD OVCAR3 and PAD1 KD SKOV3 cells, compared to the respective shRNA control cells. GAPDH served as the loading control.

**D)** Western blot analysis of p-AKT2 (S474) and p-AKT1 (S473) in HEK293 cells overexpressing AKT2 WT or R202K mutant, together with Flag-PAD1 WT or CS. GAPDH served as the loading control.

**E)** Dot blot analysis of anti-AKT2 Cit202 in detection of synthetic peptide AKT2 Cit202 and R202. Rb#1,2 representing the antibodies from the two rabbits immunized with citrullinated AKT2 peptide.

**F)** AKT2 R202 exhibiting a high level of conservation across multiple species.

Results are presented as mean ± SD, n = 3. ***p* < 0.01. A: Student’s t-test.

**Figure S5**


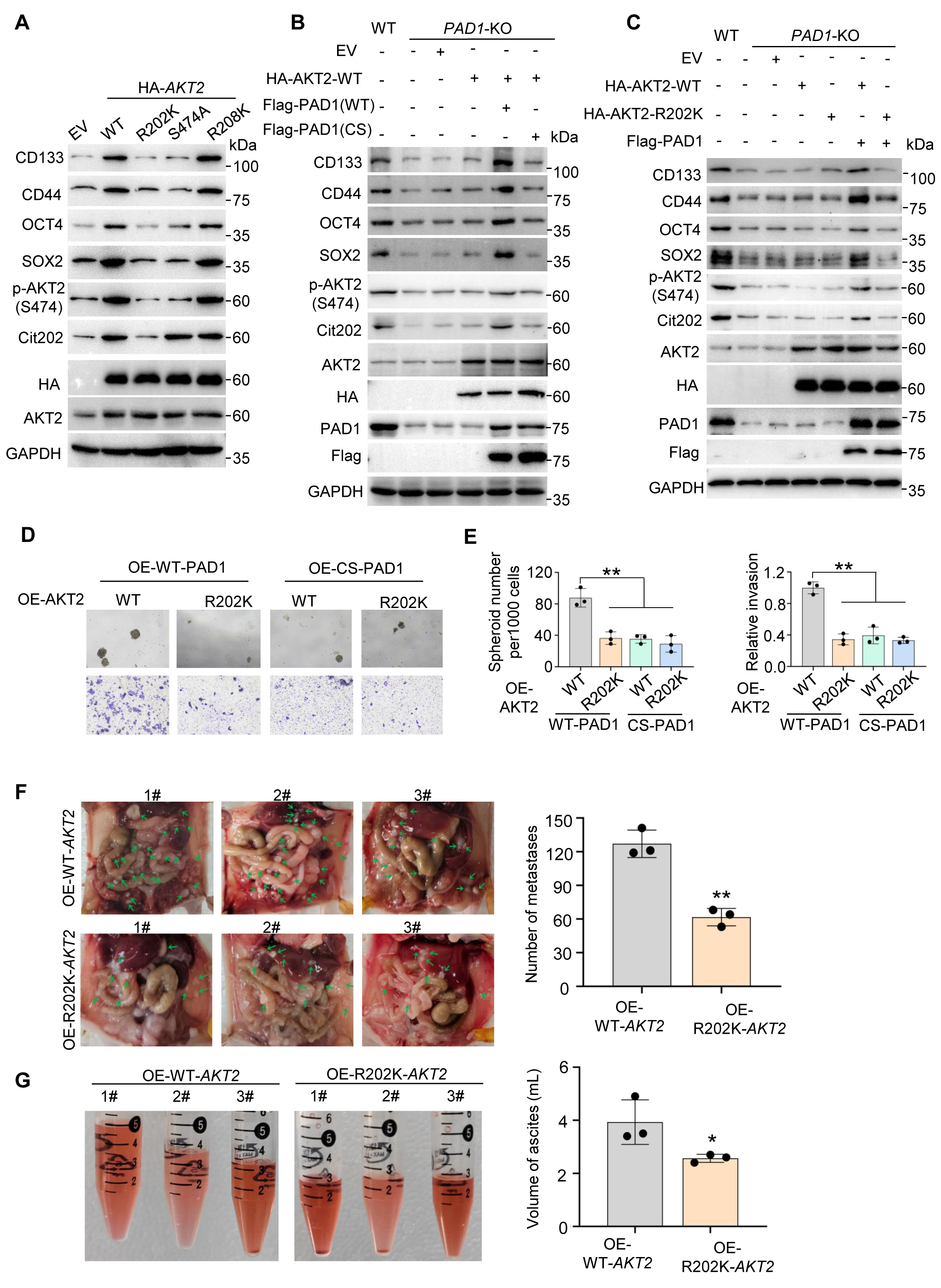


**Figure S5 Related to Figure 6**

**A)** Western blot analyses of CD133, CD44, OCT4, SOX2, p-AKT2(S474), Cit202, HA, AKT2 and GAPDH in OVCAR3 cells overexpressing AKT2 or its mutants. EV represents the empty vector control.

**B)** Western blot analyses of CD133, CD44, OCT4, SOX2, p-AKT2(S474), Cit202, AKT2, HA, PAD1, Flag and GAPDH in PAD1-KO OVCAR3 cells overexpressing Flag-PAD1 (WT or CS) and HA-WT-AKT2. EV represents the empty vector control.

**C)** Western blot analyses of CD133, CD44, OCT4, SOX2, p-AKT2(S474), Cit202, AKT2, HA, PAD1, Flag and GAPDH in OVCAR3 cells overexpressing Flag-WT-PAD1 and HA-AKT2 (WT or R202K). EV represents the empty vector control.

**D)** Representative images of spheroids derived from PAD1-KO OVCAR3 cells overexpressing PAD1 (WT or CS) and AKT2 (WT or R202K) (top), and cells in transwell assay (bottom). EV represents the empty vector.

**E)** Quantification of the spheroids with diameters greater than 100 μm (D top), and the relative invasion ability of OVCAR3 cells (D, bottom).

**F)** Intraperitoneal xenograft tumor formation in nude mice derived from AKT2 (WT or R202K) overexpressed OVCAR3 cells. The arrow indicates macroscopically visible liver, peritoneal and mesenteric metastasis (Left). Statistical analysis was performed on the number of metastasis (Right).

**G)** The volume of ascites (Left) and statistical analysis (Right) from the mice treated as described in Figure F.

Results are presented as mean ± SD, n = 3. **p* <0.05, ***p* < 0.01. D, E: one-way ANOVA; F, G: Student’s t-test.

**Figure S6**

**
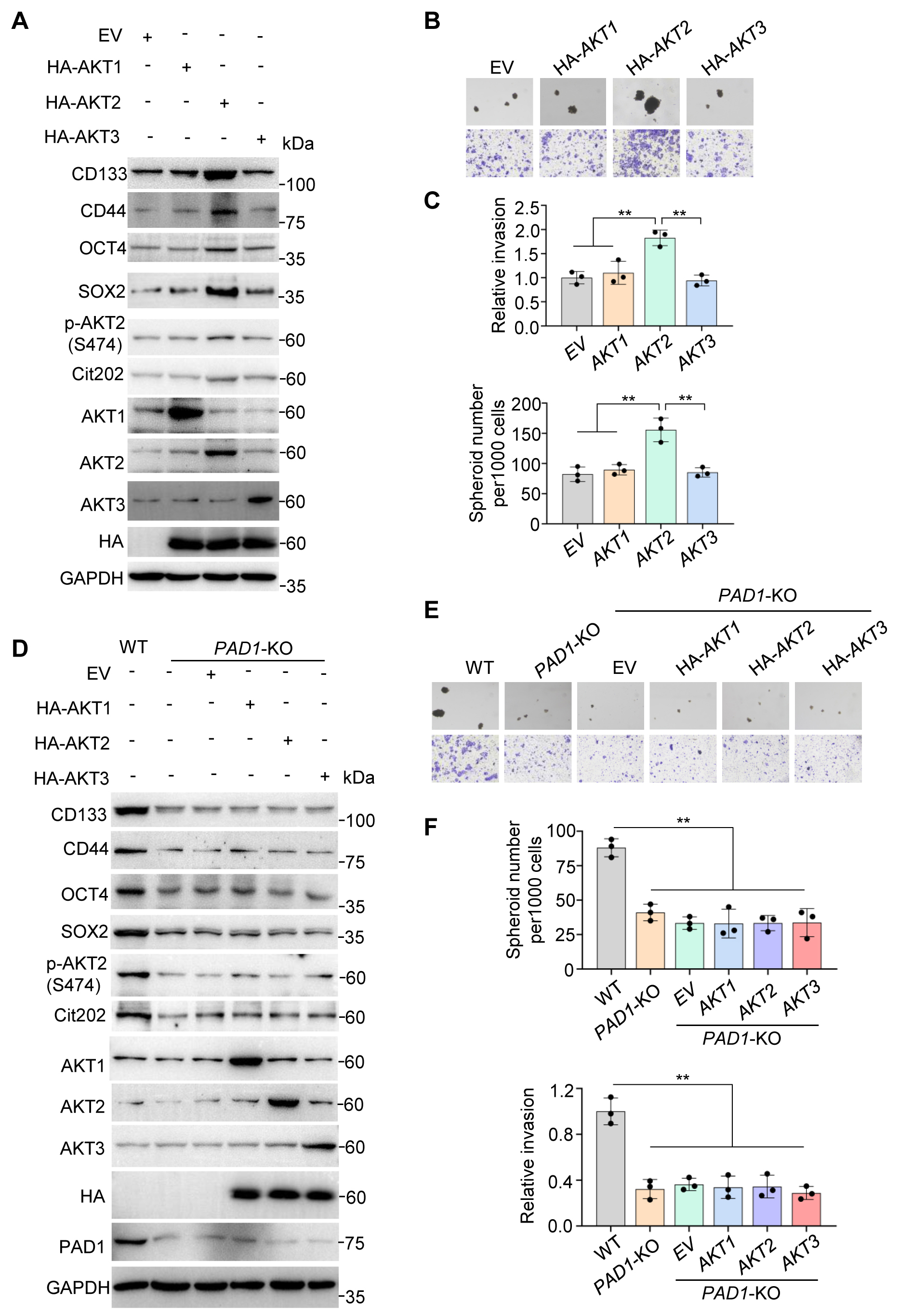
**

**Figure S6 Related to Figure 6**

**A)** Western blot analyses of CD133, CD44, OCT4, SOX2, p-AKT2(S474), Cit202, AKT1, AKT2, AKT3, HA, and GAPDH in OVCAR3 cells overexpressing AKT1, AKT2, or AKT3. EV represents the empty vector control.

**B)** Representative images of spheroids derived from OVCAR3 cells overexpressing AKT1, AKT2, or AKT3 (top), and cells in transwell assay (bottom). EV represents the empty vector.

**C)** Quantification of the spheroids with diameters greater than 100 μm (B, top), and the relative invasion ability of OVCAR3 cells (C, bottom).

**D)** Western blot analyses of CD133, CD44, OCT4, SOX2, p-AKT2(S474), Cit202, AKT1, AKT2, AKT3, HA, PAD1, and GAPDH in PAD1-KO OVCAR3 cells overexpressing AKT1, AKT2, or AKT3. EV represents the empty vector control.

**E)** Representative images of spheroids derived from PAD1-KO OVCAR3 cells overexpressing AKT1, AKT2, or AKT3 (top), and cells in transwell assay (bottom). EV represents the empty vector.

**F)** Quantification of the spheroids with diameters greater than 100 μm (E, top), and the relative invasion ability of OVCAR3 cells (E, bottom).

Results are presented as mean ± SD, n = 3. ***p* < 0.01. C, F: one-way ANOVA.

**Figure S7**

**
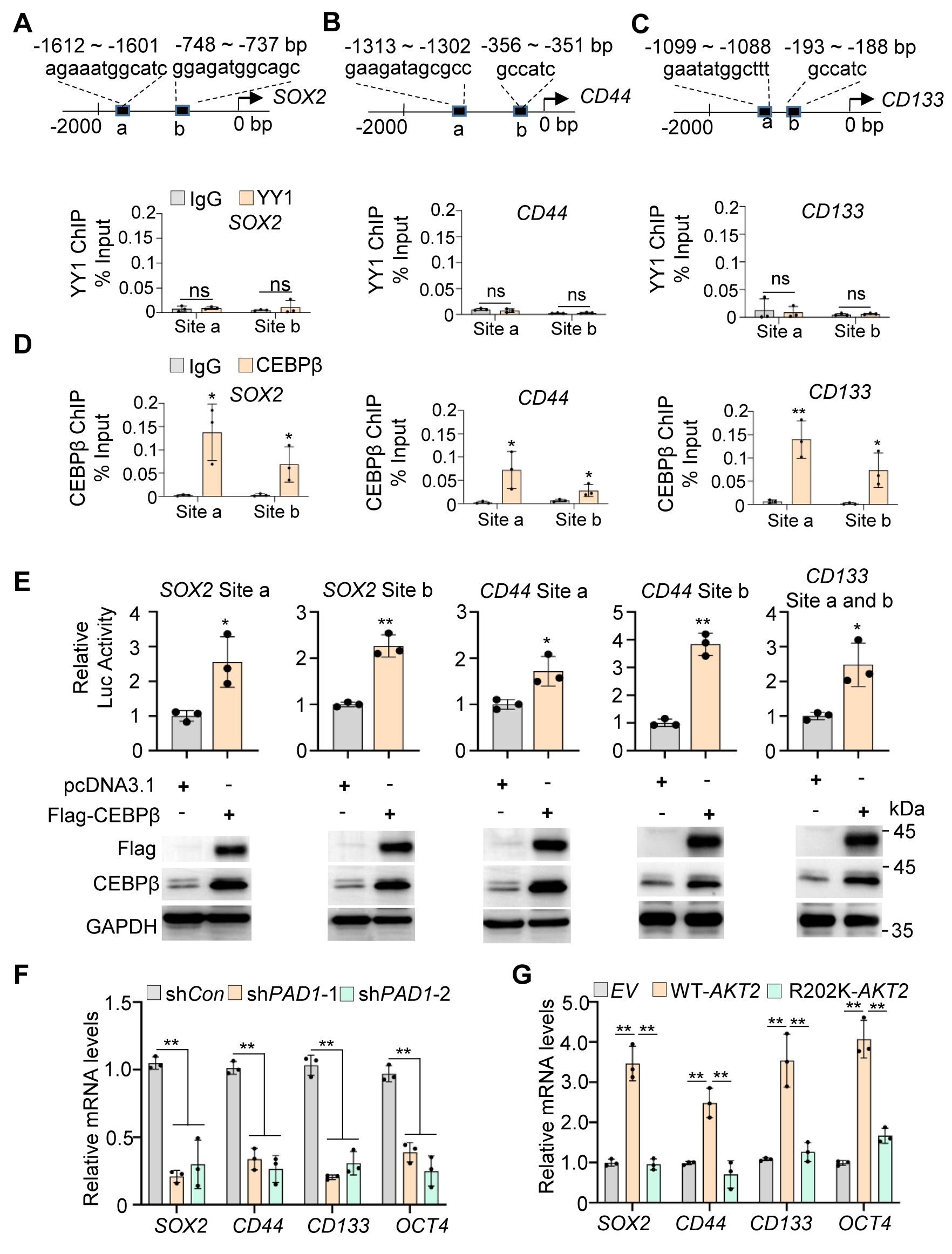
**

**Figure S7 Related to Figure 7**

**A-C)** Top: The schematic diagram indicates the locations of the YY1 binding motifs relative to the TSS for *SOX2* (A), *CD44* (B), and *CD133* (C) used for ChIP assay. Bottom: ChIP-qPCR analysis of YY1 binding at *SOX2* (A), *CD44* (B), and *CD133* (C) promoters in OVCAR3 cells. IgG was used as a control.

**D)** ChIP-qPCR analysis of CEBPβ binding at *SOX2*, *CD44*, and *CD133* promoters in OVCAR3 cells. IgG was used as a control.

**E)** HEK293 cells were co-transfected with Flag-CEBPβ and luciferase reporter constructs containing the proximal promoters of *SOX2*, *CD44*, or *CD133* with CEBPβ binding sites, separately. Reporter activity was determined relative to cells transfected with pcDNA3.1 control (top). The expression of CEBPβ was confirmed by WB with anti-Flag and anti-CEBPβ antibodies. GAPDH served as loading controls (bottom).

**F, G)** qRT-PCR analysis of *SOX2*, *CD44*, *CD133*, and *OCT4* mRNA levels in OVCAR3 cells upon PAD1 knockdown (F), and in OVCAR3 cells overexpressed HA-AKT2 (WT or R202K) (G). EV represents the empty vector control. Results are presented as mean ± SD, n = 3. **p* <0.05, ***p* < 0.01. A-E: Student’s t-test. F, G: one-way ANOVA.

**Figure S8**

**
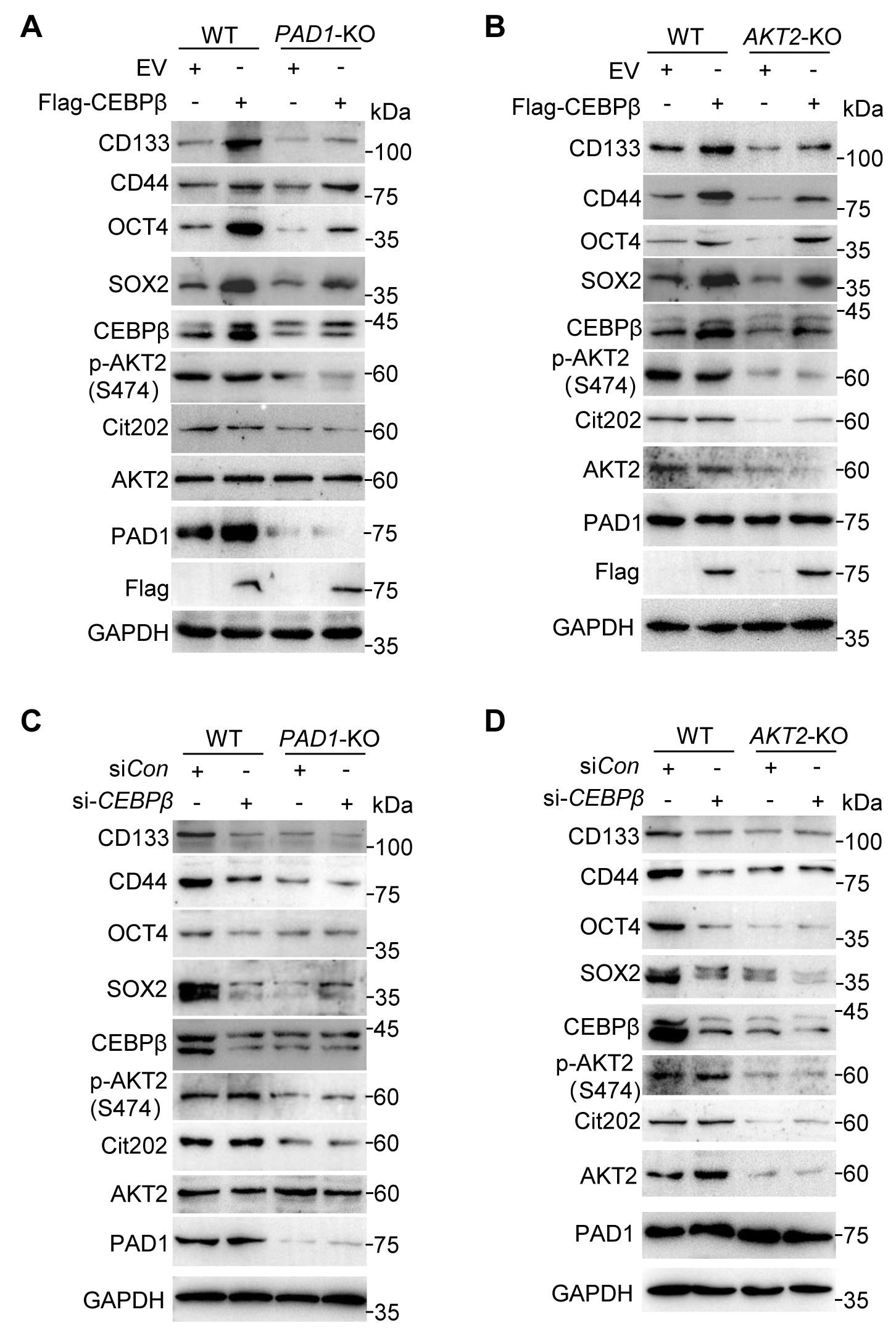
**

**Figure S8 Related to Figure 7**

**A)** Western blot analyses of CD133, CD44, OCT4, SOX2, CEBPβ, p-AKT2(S474), Cit202, AKT2, PAD1, Flag and GAPDH in OVCAR3 cells (WT or PAD1-KO) overexpressed CEBPβ. EV represents the empty vector control.

**B)** Western blot analyses of CD133, CD44, OCT4, SOX2, CEBPβ, p-AKT2(S474), Cit202, AKT2, PAD1, Flag and GAPDH in OVCAR3 cells (WT or AKT2-KO) overexpressed CEBPβ. EV represents the empty vector control.

**C)** Western blot analyses of CD133, CD44, OCT4, SOX2, CEBPβ, p-AKT2(S474), Cit202, AKT2, PAD1 and GAPDH in OVCAR3 cells (WT or PAD1-KO) knocking down CEBPβ. Si*Con* represents siRNA control.

**D)** Western blot analyses of CD133, CD44, OCT4, SOX2, CEBPβ, p-AKT2(S474), Cit202, AKT2, PAD1 and GAPDH in OVCAR3 cells (WT or AKT2-KO) knocking down CEBPβ. Si*Con* represents siRNA control.

**Figure S9**

**
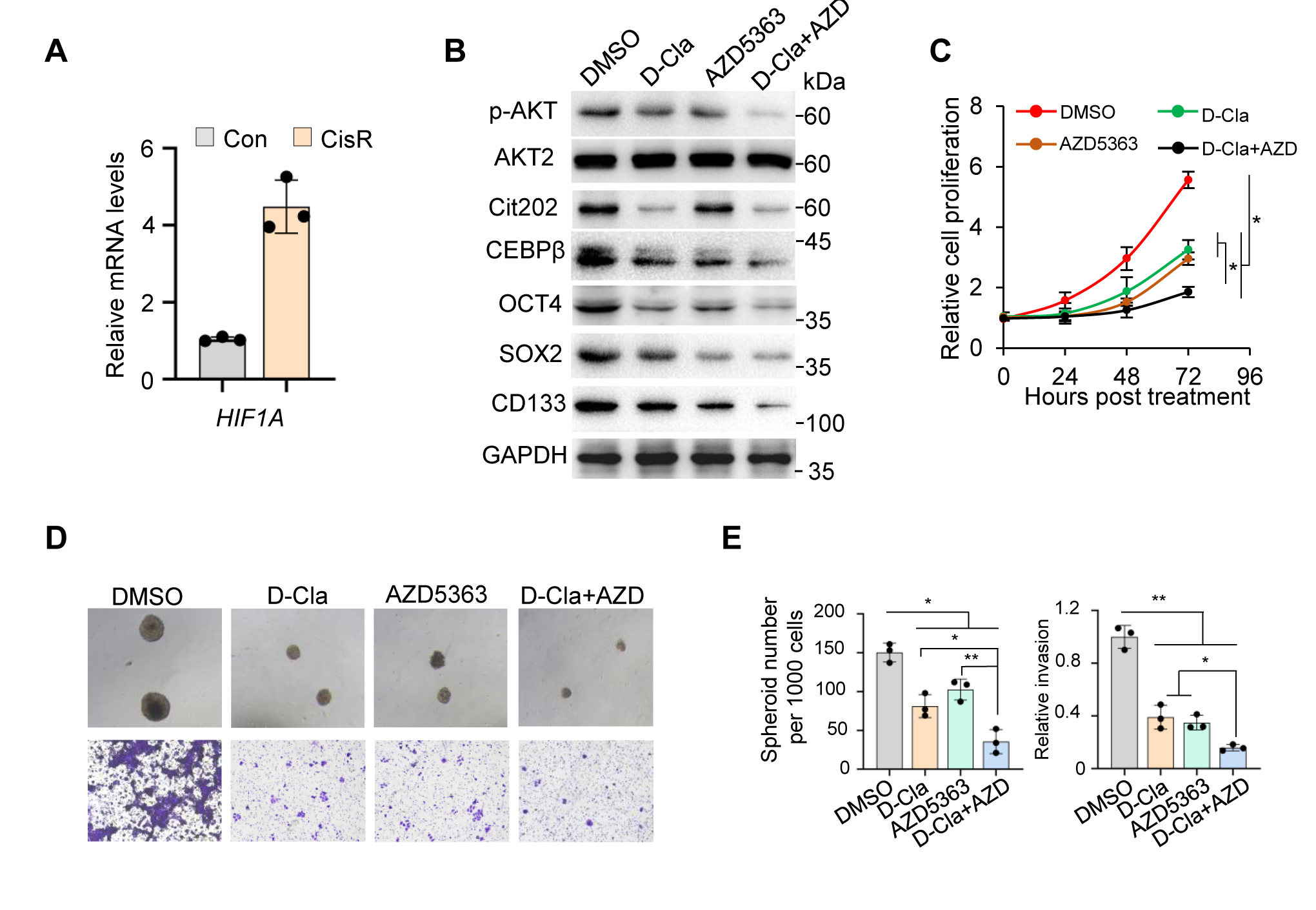
**

**Figure S9**  **Related to Figure 8**

1. Relative mRNA levels of *HIF1A* were detected in OVCAR3 control and CisR Cells by RT-qPCR. *β-Actin* was used as the reference control.

**B)** Western blot analysis of OVCAR3 CisR cells treated with D-Cla and AZD5363 using anti-p-AKT, anti-AKT2, anti-Cit202, anti-CEBPβ, anti-OCT4, anti-SOX2, and anti-CD133 antibodies. GAPDH served as loading control.

**C)** Relative cell proliferation with CCK8 assay in OVCAR3 CisR cells treated with100 µM D-Cla, 5 µM AZD5363 or the combined treatment with D-Cla and AZD.

**D, E)** Representative images (D) and quantification (E) for OVCAR3 CisR cell-derived spheroids with diameters greater than 100 μm cultured in stem cell culture medium in concave ultra-low attachment plates without FBS (top) and transwell assay (bottom) treated with D-Cla and AZD5363. Results are presented as mean ± SD, n = 3. **p* <0.05, ***p* < 0.01. A: Student’s t-test. C, E: one-way ANOVA.

**Supplementary Table 1. Antibodies used in this study**

| Antibodies | Source | Identifier |
| --- | --- | --- |
| Anti-AKT2 | Cell Signaling Technology | (L79B2) Mouse mAb #5239 |
| Anti-AKT1 | Cell Signaling Technology | (C73H10) Rabbit mAb #2938 |
| Anti-AKT3 | Cell Signaling Technology | (62A8) Rabbit mAb #3788 |
| Anti-p-AKT(T308) | Cell Signaling Technology | (D25E6) XP® Rabbit mAb #13038 |
| Anti-p-AKT(S473) | Cell Signaling Technology | (D9E) XP® Rabbit mAb  #4060 |
| Anti-p-AKT1(S473) | Cell Signaling Technology | (D7F10) XP® Rabbit mAb #9018 |
| Anti-p-AKT2(S474) | Cell Signaling Technology | (D3H2) Rabbit mAb #8599 |
| Anti-AKT | Cell Signaling Technology | #9272 |
| Anti-GAPDH | Abcam | #ab8245 |
| Anti-CD133 | Abcam | #ab19898 |
| Anti-CD44 | Abcam | #ab189524 |
| Anti-OCT4 | Abcam | #ab19857 |
| Anti-SOX2 | Abcam | #ab97959 |
| Anti-Nanog | Proteintech | #67255-1-Ig |
| Anti-CEBPβ | Abcam | #ab32358 |
| Anti-YY1 | Proteintech | #22156-1-AP |
| Anti-Flag | Sigma | #F1804 |
| Anti-HA | Yeasen | #30701ES60 |
| Anti-AKT2-Cit202 | AtaGenix Biotechnology | Not commercially available |
| Anti-PAD1 | Abcam | #ab181762 |
| Anti-PAD2 | Abcam | #ab16478 |
| Anti-PAD3 | Abcam | #ab172959 |
| Anti-PAD4 | Abcam | #ab214810 |
| Anti-CD133 for Flow CytoMetry | BD Biosciences | W6B3C1 (also known as W6B3) (RUO), #566593 |
| Goat anti-Rabbit IgG,  HRP-linked | Best Biological | #BK0027 |
| Goat anti-Mouse IgG,  HRP-linked | Best Biological | #BK0023 |
| Anti-Rabbit IgG for IP (HRP-linked) | Vazyme | #RA1008 |
| Anti-Mouse IgG for IP (HRP-linked) | Vazyme | #RA1009 |
| Goat anti-Mouse IgG,  Alexa Fluor 488 linked | Invitrogen | #A-11001 |
| Goat anti-Mouse IgG,  Alexa Fluor 546 linked | Invitrogen | #A-11035 |
| Normal Rabbit IgG | Millipore | #12-370 |

**Su**pplementary Table 2. Primers for RT-qPCR

| **Gene** | **Primer** | **Sequence (5′-3′)** |
| --- | --- | --- |
| *PAD1* | Forward | GAGTGATGGACACTCATGGC |
|  | Reverse | CAGATGGTCAGCTTGCAGTT |
| *PAD2* | Forward | TCTCAGGCCTGGTCTCCAT |
|  | Reverse | AAGATGGGAGTCAGGGGAAT |
| *PAD3* | Forward | AGCAATGACCTCAACGACAG |
|  | Reverse | TGAGGTAGAGCACCGCATAG |
| *PAD4* | Forward | TCACCTACCACATCAGGCAT |
|  | Reverse | CATGTTCCACCACTTGAAGG |
| *CD133* | Forward | ACGCACAGGGAATGGATTGT |
|  | Reverse | GGTTTGCACGATGCCACTTT |
| *CD44* | Forward | CTGCCGCTTTGCAGGTGTA |
|  | Reverse | CATTGTGGGCAAGGTGCTATT |
| *OCT4* | Forward | GCTCGAGAAGGATGTGGTCC |
|  | Reverse | CGTTGTGCATAGTCGCTGCT |
| *SOX2* | Forward | AACCAGCGCATGGACAGTTA |
|  | Reverse | CGAGCTGGTCATGGAGTTGT |
| *ABCB1* | Forward | GTGGGGCAAGTCAGTTCATT |
|  | Reverse | TCTTCACCTCCAGGCTCAGT |
| *ABCC1* | Forward | TTACTCATTCAGCTCGTCTTGTC |
|  | Reverse | CAGGGATTAGGGTCGTGGAT |
| *ABCG2* | Forward | GGGTTCTCTTCTTCCTGACGACC |
|  | Reverse | TGGTTGTGAGATTGACCAACAGACC |
| *HIF1A* | Forward | GTCTGAGGGGACAGGAGGAT |
|  | Reverse | CTCCTCAGGTGGCTTGTCAG |
| *GAPDH* | Forward | GAAATCCCATCACCATCTTCCAGG |
|  | Reverse | GAGCCCCAGCCTTCTCCATG |
| *β-Actin* | Forward | CATGTACGTTGCTATCCAGGC |
|  | Reverse | CTCCTTAATGTCACGCACGAT |

**Su**pplementary Table 3. Primers for ChIP-qPCR

| **Gene** | **Primer** | **Sequence (5′-3′)** |
| --- | --- | --- |
| *SOX2*-CEBPβ-Site a | Forward | ACTTTTCTGAGTTTCCAGTGGGT |
|  | Reverse | CCGACCCCCAATTTCTCCAA |
| *SOX2*-CEBPβ-Site b | Forward | GTGGGATGCCAGGAAGTTGA |
|  | Reverse | CATGCCTCCCCGTAAGAAGG |
| *CD44*-CEBPβ-Site a | Forward | AGGCAAGAAGTCCATGCAGA |
|  | Reverse | AGGGGAAGCCTTTTGAGATTGA |
| *CD44*-CEBPβ-Site b | Forward | ATCTTGCTCCAGCCGGATTC |
|  | Reverse | GGACAGAGGATGACCGAACC |
| *CD133*-CEBPβ-Site a | Forward | CACAGAGCGGGAAGACCAAT |
|  | Reverse | CTAGTGGCTGAAGCGGTTGA |
| *CD133*-CEBPβ-Site b | Forward | TCAACCGCTTCAGCCACTAG |
|  | Reverse | GCCGCATTAGACCCTTCTGT |
| *SOX2*-YY1-Site a | Forward | GGGCGGAGAGAGTGTTACAG |
|  | Reverse | GACTACAAAGGGTGGGGTGG |
| *SOX2*-YY1-Site b | Forward | TGGGGCTAAGAGGAAGAGCT |
|  | Reverse | GGTAAGCTGCCATCTCCCTG |
| *CD44*-YY1-Site a | Forward | AGGGTGAGGGCTCTGAAGAT |
|  | Reverse | CAGCCATCCCCCTATGCATT |
| *CD44*-YY1-Site b | Forward | ACACATGGGTTAGCTGAGCC |
|  | Reverse | TCTTGGCAGAACAGCTCAGG |
| *CD133*-YY1-Site a | Forward | CACAGAGCGGGAAGACCAAT |
|  | Reverse | CTAGTGGCTGAAGCGGTTGA |
| *CD133-*YY1-Site b | Forward | GAGAGAGGCATCTGCTGACC |
|  | Reverse | CGCAGATGGCTAGGGTAAGG |

**Supplementary Table 4. **Oligonucleotides for generating point mutation****

| **Gene** | **Primer** | **Sequence (5′-3′)** |
| --- | --- | --- |
| AKT2 R202E | Forward | CAGTCACCGAGAGCGAGGTCCTCCAGAACACC |
|  | Reverse | GGTGTTCTGGAGGACCTCGCTCTCGGTGACTG |
| AKT2 R202K | Forward | CAGTCACCGAGAGCAAGGTCCTCCAGAACACC |
|  | Reverse | GGTGTTCTGGAGGACCTTGCTCTCGGTGACTG |
| AKT2 R202A | Forward | CAGTCACCGAGAGCGCGGTCCTCCAGAACACC |
|  | Reverse | GGTGTTCTGGAGGACCGCGCTCTCGGTGACTG |
| AKT2 R208E | Forward | CCTCCAGAACACCGAGCACCCGTTCCTC |
|  | Reverse | GAGGAACGGGTGCTCGGTGTTCTGGAGG |
| AKT2 R208K | Forward | CCTCCAGAACACCAAGCACCCGTTCCTC |
|  | Reverse | GAGGAACGGGTGCTTGGTGTTCTGGAGG |
| AKT2 R208A | Forward | CCTCCAGAACACCGCGCACCCGTTCCTC |
|  | Reverse | GAGGAACGGGTGCGCGGTGTTCTGGAGG |
| AKT2 R224E | Forward | CCAGACCCACGACGAGCTGTGCTTTGTGATGG |
|  | Reverse | CCATCACAAAGCACAGCTCGTCGTGGGTCTGG |
| AKT2 R224K | Forward | CCAGACCCACGACAAGCTGTGCTTTGTGATGG |
|  | Reverse | CCATCACAAAGCACAGCTTGTCGTGGGTCTGG |
| AKT2 R224A | Forward | CCAGACCCACGACGCCCTGTGCTTTGTGATGG |
|  | Reverse | CCATCACAAAGCACAGGGCGTCGTGGGTCTGG |
| AKT2 R371E | Forward | AGATCCGCTTCCCGGAA ACGCTCAGCCCCGAGG |
|  | Reverse | CCTCGGGGCTGAGCGTTTCCGGGAAGCGGATCT |
| AKT2 R371K | Forward | AGATCCGCTTCCCGAAA ACGCTCAGCCCCGAGG |
|  | Reverse | CCTCGGGGCTGAGCGTTTTCGGGAAGCGGATCT |
| AKT2 R371A | Forward | AGATCCGCTTCCCGGCCACGCTCAGCCCCGAGG |
|  | Reverse | CCTCGGGGCTGAGCGTGGCCGGGAAGCGGATCT |
| AKT2 S474A | Forward | CCAGTTCGCCTACTCGGCCAGCATCCGCGAGTG |
|  | Reverse | GGTCAAGCGGATGAGCCGGTCGTAGGCGCTCAC |

**Supplementary Table 5. The shRNA, siRNA and** CRISPR sgRNA sequences

| Gene | Primer | Sequence (5’-3’) |
| --- | --- | --- |
| sh*PAD1-1* | Forward | GATCCAGGTGAGGGTCTCCTACTTTGTTCAAGAGACAAAGTAGGAGACCCTCACCTTTTTTTG |
|  | Reverse | AATTCAAAAAAAGGTGAGGGTCTCCTACTTTGTCTCTTGAACAAAGTAGGAGACCCTCACCTG |
| sh*PAD1-2* | Forward | GATCCAGAGGCTCTAGATCAACAATGTTCAAGAGACATTGTTGATCTAGAGCCTCTTTTTTTG |
|  | Reverse | AATTCAAAAAAAGAGGCTCTAGATCAACAATGTCTCTTGAACATTGTTGATCTAGAGCCTCTG |
| si*Con* | sense | UUCUCCGAACGUGUCACGUdTdT |
|  | antisense | ACGUGACACGUUCGGAGAAdTdT |
| si*CEBPβ-1* | sense | AGCACAGCGACGAGUACAAdGdA |
|  | antisense | UUGUACUCGUCGCUGUGCUdTdG |
| si*CEBPβ-2* | sense | CCCUGCGGAACUUGUUCAAdGdC |
|  | antisense | UUGAACAAGUUCCGCAGGGdTdG |
| si*CEBPβ-3* | sense | ACCUCUUCUCCGACGACUAdCdG |
|  | antisense | UAGUCGUCGGAGAAGAGGUdCdG |
| sg*PAD1-1* | | CATCAGTGTCTAGCGGCCAA |
| sg*PAD1-2* | | TGCAGACATGGTCGTATCTG |
| sg*PAD1-3* | | CAGTGAGGTAAAGCACGCTG |
| sg*AKT2-1* | | acatcaagacctggaggcca |
| sg*AKT2-2* | | AGAGCGACGGCTCCTTCATT |
| sg*AKT2-3* | | GAGCCACACTTGTAGTCCAT |

**Supplementary Table 6. Tumor weight and corresponding animal weight (g)**

| **Figure 1H** | **Weight of tumor (g)** | **Weight of animal (g)** |
| --- | --- | --- |
| #01-R (shCon) | 0.521 | 22.513 |
| #01-L (shPAD1) | 0.112 |
| #02-R (shCon) | 0.796 | 21.656 |
| #02-L (shPAD1) | 0.082 |
| #03-R (shCon) | 0.658 | 21.841 |
| #03-L (shPAD1) | 0.134 |
| #04-R (shCon) | 0.583 | 20.957 |
| #04-L (shPAD1) | 0.181 |
| #05-R (shCon) | 0.742 | 22.131 |
| #05-L (shPAD1) | 0.077 |
| #06-R (shCon) | 0.615 | 22.245 |
| #06-L (shPAD1) | 0.081 |
|  | | |
| **Figure 2C** | **Weight of tumor (g)** | **Weight of animal (g)** |
| #01-R (shCon 1×10^7^) | 0.812 | 23.563 |
| #01-L (shCon 1×10^7^) | 0.791 |
| #02-R (shCon 1×10^7^) | 0.748 | 22.752 |
| #02-L (shCon 1×10^7^) | 0.703 |
| #03-R (shCon 1×10^7^) | 0.614 | 23.116 |
| #03-L (shCon 1×10^7^) | 0.681 |
| #04-R (shPAD1 1×10^7^) | 0.485 | 23.023 |
| #04-L (shPAD1 1×10^7^) | 0.295 |
| #05-R (shPAD1 1×10^7^) | 0.246 | 22.944 |
| #05-L (shPAD1 1×10^7^) | 0.215 |
| #06-R (shPAD1 1×10^7^) | 0.233 | 23.222 |
| #06-L (shPAD1 1×10^7^) | 0.321 |
| #07-R (shCon 1×10^6^) | 0.711 | 22.183 |
| #07-L (shCon 1×10^6^) | 0.641 |
| #08-R (shCon 1×10^6^) | 0.657 | 21.192 |
| #08-L (shCon 1×10^6^) | 0.534 |
| #09-R (shCon 1×10^6^) | 0.561 | 22.274 |
| #09-L (shCon 1×10^6^) | 0.000 |
| #10-R (shPAD1 1×10^6^) | 0.231 | 21.893 |
| #10-L (shPAD1 1×10^6^) | 0.192 |
| #11-R (shPAD1 1×10^6^) | 0.244 | 22.087 |
| #11-L (shPAD1 1×10^6^) | 0.000 |
| #12-R (shPAD1 1×10^6^) | 0.000 | 21.558 |
| #12-L (shPAD1 1×10^6^) | 0.000 |
| #13-R (shCon 1×10^5^) | 0.214 | 21.962 |
| #13-L (shCon 1×10^5^) | 0.191 |
| #14-R (shCon 1×10^5^) | 0.144 | 22.019 |
| #14-L (shCon 1×10^5^) | 0.000 |
| #15-R (shCon 1×10^5^) | 0.000 | 21.991 |
| #15-L (shCon 1×10^5^) | 0.000 |
| #16-R (shPAD1 1×10^5^) | 0.064 | 22.074 |
| #16-L (shPAD1 1×10^5^) | 0.000 |
| #17-R (shPAD1 1×10^5^) | 0.000 | 21.265 |
| #17-L (shPAD1 1×10^5^) | 0.000 |
| #18-R (shPAD1 1×10^5^) | 0.000 | 23.044 |
| #18-L (shPAD1 1×10^5^) | 0.000 |
| #19-R (shCon 1×10^4^) | 0.054 | 22.516 |
| #19-L (shCon 1×10^4^) | 0.000 |
| #20-R (shCon 1×10^4^) | 0.000 | 21.827 |
| #20-L (shCon 1×10^4^) | 0.000 |
| #21-R (shCon 1×10^4^) | 0.000 | 22.332 |
| #21-L (shCon 1×10^4^) | 0.000 |
| #22-R (shPAD1 1×10^4^) | 0.000 | 21.840 |
| #22-L (shPAD1 1×10^4^) | 0.000 |
| #23-R (shPAD1 1×10^4^) | 0.000 | 22.731 |
| #23-L (shPAD1 1×10^4^) | 0.000 |
| #24-R (shPAD1 1×10^4^) | 0.000 | 21.643 |
| #24-L (shPAD1 1×10^4^) | 0.000 |
|  | | |
| **Figure 4I** | **Weight of tumor (g)** | **Weight of animal (g)** |
| #01-R (PBS) | 0.651 | 22.142 |
| #01-L (D-Cla) | 0.230 |
| #02-R (PBS) | 0.452 | 21.613 |
| #02-L (D-Cla) | 0.191 |
| #03-R (PBS) | 0.760 | 21.828 |
| #03-L (D-Cla) | 0.150 |
| #04-R (PBS) | 0.893 | 22.031 |
| #04-L (D-Cla) | 0.132 |
| #05-R (PBS) | 0.431 | 20.992 |
| #05-L (D-Cla) | 0.175 |
| #06-R (PBS) | 0.373 | 21.224 |
| #06-L (D-Cla) | 0.091 |
|  | | |
| **Figure 6F** | **Weight of tumor (g)** | **Weight of animal (g)** |
| #01-R (WT AKT2 1×10^7^) | 0.850 | 21.012 |
| #01-L (WT AKT2 1×10^7^) | 0.537 |
| #02-R (WT AKT2 1×10^7^) | 0.794 | 22.044 |
| #02-L (WT AKT2 1×10^7^) | 0.452 |
| #03-R (WT AKT2 1×10^7^) | 0.491 | 20.223 |
| #03-L (WT AKT2 1×10^7^) | 0.512 |
| #04-R (R202K AKT2 1×10^7^) | 0.313 | 21.327 |
| #04-L (R202K AKT2 1×10^7^) | 0.283 |
| #05-R (R202K AKT2 1×10^7^) | 0.239 | 20.144 |
| #05-L (R202K AKT2 1×10^7^) | 0.207 |
| #06-R (R202K AKT2 1×10^7^) | 0.215 | 21.517 |
| #06-L (R202K AKT2 1×10^7^) | 0.223 |
| #07-R (WT AKT2 1×10^6^) | 0.347 | 20.665 |
| #07-L (WT AKT2 1×10^6^) | 0.324 |
| #08-R (WT AKT2 1×10^6^) | 0.292 | 20.847 |
| #08-L (WT AKT2 1×10^6^) | 0.215 |
| #09-R (WT AKT2 1×10^6^) | 0.313 | 20.953 |
| #09-L (WT AKT2 1×10^6^) | 0.292 |
| #10-R (R202K AKT2 1×10^6^) | 0.257 | 21.152 |
| #10-L (R202K AKT2 1×10^6^) | 0.201 |
| #11-R (R202K AKT2 1×10^6^) | 0.190 | 20.194 |
| #11-L (R202K AKT2 1×10^6^) | 0.184 |
| #12-R (R202K AKT2 1×10^6^) | 0.173 | 21.535 |
| #12-L (R202K AKT2 1×10^6^) | 0.000 |
| #13-R (WT AKT2 1×10^5^) | 0.125 | 21.341 |
| #13-L (WT AKT2 1×10^5^) | 0.217 |
| #14-R (WT AKT2 1×10^5^) | 0.116 | 20.993 |
| #14-L (WT AKT2 1×10^5^) | 0.000 |
| #15-R (WT AKT2 1×10^5^) | 0.000 | 21.602 |
| #15-L (WT AKT2 1×10^5^) | 0.000 |
| #16-R (R202K AKT2 1×10^5^) | 0.105 | 20.991 |
| #16-L (R202K AKT2 1×10^5^) | 0.071 |
| #17-R (R202K AKT2 1×10^5^) | 0.000 | 21.884 |
| #17-L (R202K AKT2 1×10^5^) | 0.000 |
| #18-R (R202K AKT2 1×10^5^) | 0.000 | 20.657 |
| #18-L (R202K AKT2 1×10^5^) | 0.000 |
| #19-R (WT AKT2 1×10^4^) | 0.041 | 21.159 |
| #19-L (WT AKT2 1×10^4^) | 0.000 |
| #20-R (WT AKT2 1×10^4^) | 0.000 | 20.851 |
| #20-L (WT AKT2 1×10^4^) | 0.000 |
| #21-R (WT AKT2 1×10^4^) | 0.000 | 21.258 |
| #21-L (WT AKT2 1×10^4^) | 0.000 |
| #22-R (R202K AKT2 1×10^4^) | 0.000 | 21.126 |
| #22-L (R202K AKT2 1×10^4^) | 0.000 |
| #23-R (R202K AKT2 1×10^4^) | 0.000 | 21.555 |
| #23-L (R202K AKT2 1×10^4^) | 0.000 |
| #24-R (R202K AKT2 1×10^4^) | 0.000 | 20.862 |
| #24-L (R202K AKT2 1×10^4^) | 0.000 |
|  | | |
| **Figure 8M** | **Weight of tumor (g)** | **Weight of animal (g)** |
| #01-R (Con/Cis) | 0.211 | 21.854 |
| #01-L (Con/Cis) | 0.292 |
| #02-R (Con/Cis) | 0.223 | 21.298 |
| #02-L (Con/Cis) | 0.390 |
| #03-R (Con/Cis) | 0.091 | 22.151 |
| #03-L (Con/Cis) | 0.130 |
| #04-R (Con/Cis) | 0.317 | 20.154 |
| #04-L (Con/Cis) | 0.110 |
| #05-R (Con/Cis) | 0.243 | 22.511 |
| #05-L (Con/Cis) | 0.234 |
| #06-R (CisR/Cis) | 0.621 | 21.587 |
| #06-L (CisR/Cis) | 0.510 |
| #07-R (CisR/Cis) | 1.101 | 22.614 |
| #07-L (CisR/Cis) | 0.989 |
| #08-R (CisR/Cis) | 0.570 | 23.001 |
| #08-L (CisR/Cis) | 0.360 |
| #09-R (CisR/Cis) | 1.141 | 22.145 |
| #09-L (CisR/Cis) | 0.751 |
| #10-R (CisR/Cis) | 0.322 | 21.532 |
| #10-L (CisR/Cis) | 0.470 |
| #11-R (CisR/Cis+D-Cla) | 0.190 | 20.157 |
| #11-L (CisR/Cis+D-Cla) | 0.233 |
| #12-R (CisR/Cis+D-Cla) | 0.110 | 20.965 |
| #12-L (CisR/Cis+D-Cla) | 0.181 |
| #13-R (CisR/Cis+D-Cla) | 0.154 | 21.357 |
| #13-L (CisR/Cis+D-Cla) | 0.120 |
| #14-R (CisR/Cis+D-Cla) | 0.210 | 22.021 |
| #14-L (CisR/Cis+D-Cla) | 0.083 |
| #15-R (CisR/Cis+D-Cla) | 0.244 | 21.365 |
| #15-L (CisR/Cis+D-Cla) | 0.171 |
